# Non-Fullerene Acceptor-Based Solar Cells: From Structural Design to Interface Charge Separation and Charge Transport

**DOI:** 10.3390/polym9120692

**Published:** 2017-12-08

**Authors:** Qungui Wang, Yuanzuo Li, Peng Song, Runzhou Su, Fengcai Ma, Yanhui Yang

**Affiliations:** 1 College of Science, Northeast Forestry University, Harbin 150040, China; qunguiwang@126.com (Q.W.); 13503631076@163.com (R.S.); 2 Department of Physics, Liaoning University, Shenyang 110036, China; songpeng@lnu.edu.cn (P.S.); fengcaima@lnu.edu.cn (F.M.); 3 School of Chemical and Biomedical Engineering, Nanyang Technological University, Singapore 639798, Singapore

**Keywords:** non-fullerene acceptors (NFAs), polymers, mobility, charge separation and transport, solar cells

## Abstract

The development of non-fullerene small molecule as electron acceptors is critical for overcoming the shortcomings of fullerene and its derivatives (such as limited absorption of light, poor morphological stability and high cost). We investigated the electronic and optical properties of the two selected promising non-fullerene acceptors (NFAs), IDIC and IDTBR, and five conjugated donor polymers using quantum-chemical method (QM). Based on the optimized structures of the studied NFAs and the polymers, the ten donor/acceptor (D/A) interfaces were constructed and investigated using QM and Marcus semi-classical model. Firstly, for the two NFAs, IDTBR displays better electron transport capability, better optical absorption ability, and much greater electron mobility than IDIC. Secondly, the configurations of D/A yield the more bathochromic-shifted and broader sunlight absorption spectra than the single moiety. Surprisingly, although IDTBR has better optical properties than IDIC, the IDIC-based interfaces possess better electron injection abilities, optical absorption properties, smaller exciton binding energies and more effective electronic separation than the IDTBR-based interfaces. Finally, all the polymer/IDIC interfaces exhibit large charge separation rate (*K*_CS_) (up to 10^12^–10^14^ s^−1^) and low charge recombination rate (*K*_CR_) (<10^6^ s^−1^), which are more likely to result in high power conversion efficiencies (PCEs). From above analysis, it was found that the polymer/IDIC interfaces should display better performance in the utility of bulk-heterojunction solar cells (BHJ OSC) than polymer/IDTBR interfaces.

## 1. Introduction

With the exhaustion of fossil fuel and the sustained environmental pollution, seeking new sustainable and clean energy is imminent. Solar energy is an ideal candidate because solar energy is clean and has tremendous reserve. Organic solar cell (OSC) is the core device for converting solar energy into electric energy, and bulk-heterojunction (BHJ) polymer solar cells play an important role in many solar cell devices [1,2,3]. In recent decades, polymer solar cells (PSCs) have attracted much attention because of their own advantages such as being a low-cost, flexible, and lightweight material, and having a large-area fabrication [4,5,6,7]. The active layer is a key part of the heterojunction solar cell, which typically consists of the electronic donor and electronic acceptor materials, installing into a bilayer structure or in the form of a blend [8], and the volume density of organic active layer has direct influence on organic photovoltaics (OPV) performance [8]. Many efforts for a more sustainable photovoltaic technology have been performed from green chemistry strategies to synthesizing conjugated organic semiconductors [9]. Meanwhile, click chemistry reaction provides an effective way to prepare and synthesize the diverse conjugated polymer and oligomer in solar cells [10]. To date, most BHJ solar cells use fullerene derivatives as the electron acceptor, the PCEs of which have exceeded 10% [7,11], because of fullerene’s high electron affinity and isotropic charge transport [12,13]. Nevertheless, fullerene’s derivatives have defects, such as weak absorption in the visible region, and high cost in their preparation and purification process [14,15,16]. These disadvantages led to a rapid development of non-fullerene solar systems [9,10,17,18,19], such as the all-polymer organic solar cells (all-PSCs) and the BHJ solar cell substituting fullerene acceptors with small molecular acceptors. Bumjoon J. Kim and coworkers have demonstrated that the system based on PBDTTTPD polymer donor and P(NDI2HD-T) polymer acceptor displayed power conversion efficiencies (PCE) of 6.64% [18], which was higher than that of polymer-fullerene system. A PCE of 10.1% for all-PSCs containing PTzBI-Si copolymer has been achieved [19]. The merit of the tunable structural design and the improvement of stabilities make all-polymer organic solar cells become favorable candidates in the real application. The non-fullerene small molecule acceptors (NFAs) have attracted many researchers’ attentions by the light of readily tunable electronic energy levels, high absorption coefficients and easy synthesis [20]. Several kinds of small-molecule acceptors have been reported (such as perylene diimide (PDI), naphthalene diimide (NDI) anddiketopyrrolopyrrole (DPP) and so on [21,22,23,24,25,26,27,28]), and the performance of some NF-based OSCs have exceeding their fullerene-based system.

Recently, the small molecule NFAs called IDTBR (which was based on an indacenodithiophene core with benzothiadiazole and rhodanine flanking groups), was synthesized, and the solar cell device based on the P3HT:IDTBR blend achieved a high PCE up to 6.4% [29]. Another new planar fused-ring NFA (IDIC) based on indacenodithiophene was designed and synthesized [30]. Based on IDIC, two systems, PDBT-T1/IDIC and P-BZS/IDIC, were optimized using 1,8-diiodooctane (DIO) as solvent additive to tune the morphology and nanoscale phase separation; use of 0.25% DIO boosts the PCE of OSCs based on the PDBT-T1:IDIC and the P-BZS:IDIC up to 10.37% and 11.03%, respectively [31].

**Figure 1:**
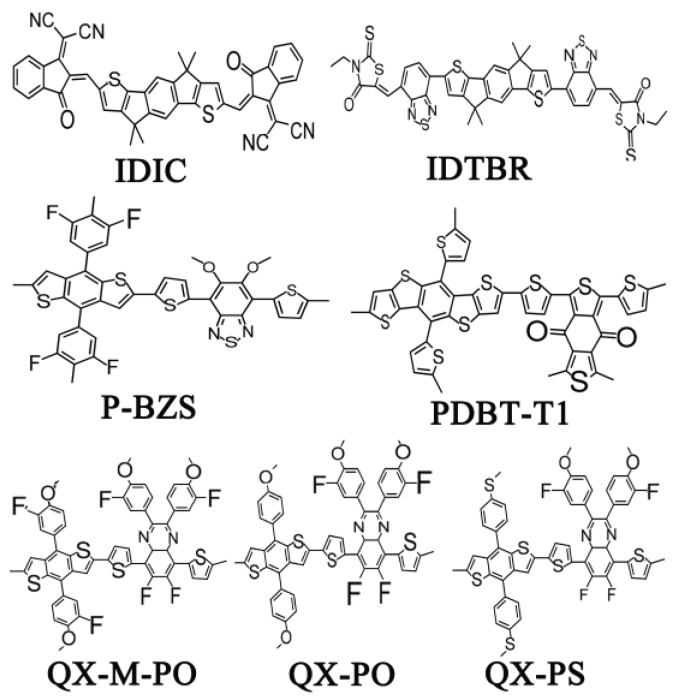
The chemical structures of Non-fullerene acceptors (NFAs) (IDTBR and IDIC) and the five kinds of polymers (P-BZS, PDBT-T1, QX-M-PO, QX-PO, and QX-PS).

Stimulated by these recent reports, we used quantum chemistry methods to study the structure, absorption, and charge transport, charge transfer in interface of PDBT-T1/IDIC and P-BZS/IDIC from the view point of theory. Further, the new interface configurations were constructed using three polymers as donor (QX-M-PO, QX-PO, and QX-PS) and NFAs (IDTBR and IDIC) to explore the potential utility in solar cell. Those polymers from tetrafluoridequidequinoxaline (ffQX) and three distinctive phenyl substituted benzodithiophene (BDT) named PffQX-m-fPO, PffQX-PO, and PffQX-PS (abbreviated as QX-M-PO, QX-PO, and QX-PS, respectively) were designed and synthesized [32]. All the polymer solar cells with these three polymers as donor exhibited higher *V_OC_* value than 0.9 V, and PCEs over 7% (7.0% for QX-M-PO, 7.4% for QX-PO and 8.0% for QX-PS). The chemical structures of IDTBR, IDIC and the five polymers (P-BZS, PDBT-T1, QX-M-PO, QX-PO, and QX-PS) are shown in Figure 1. Based on the optimized geometry structures, we constructed ten donor/acceptor (D/A) interfaces, which include the two manufactured D/A interfaces (P-BZS/IDIC and PDBT-T1/IDIC) and eight newly designed D/A interfaces (QX-M-PO/IDIC, QX-PO/IDIC, QX-PS/IDIC, P-BZS/IDTBR, PDBT-T1/IDTBR, QX-M-PO/IDTBR, QX-PO/IDTBR, and QX-PS/IDTBR). The aim of the current work is to identify the performance of interface by means of the same polymers coupled with the NFAs. From calculation, we obtained the diverse properties, such as the ground state structures, the highest occupied molecular orbitals (HOMOs), the lowest unoccupied molecular orbitals (LUMOs), ionization potentials (IPs), electron affinities (EAs), spectra, electron and hole reorganization energies, and electron and hole mobilities. For ten D/A interfaces, some important parameters affecting the PCE of OSC were evaluated and analyzed, such as the energy levels related to the open-circuit voltage (*V_OC_*) [33,34], the absorption spectra related to light-absorbing efficiency [35], the charge separation/recombination rates related to short-circuit current density (*J_SC_*) [36], etc.

## 2. Computational Methods

First, the ground-state geometry structures of the NFAs (IDTBR and IDIC) and the five polymers (P-BZS, PDBT-T1, QX-M-PO, QX-PO, and QX-PS) have been optimized by using density functional theory (DFT) method [37] with B3LYP functional [38] at the 6-31G(d) basis set. All the side alkyl chains of molecules were replaced by the methyl for saving the computational resources and time, since it was confirmed that the side alkyl chain has little influence on the electronic structures and optical properties of the materials [39]. Afterwards, based on the optimized ground-state geometry structures, the absorption spectra were calculated by time-dependent density functional theory (TD-DFT) method [40] at CAM-B3LYP functional [41] at the 6-31G(d) level. To study the reorganization energies, the cationic/anionic geometries of IDTBR and IDIC and the five polymers were also optimized with DFT/B3LYP/6-31G(d).

For the D/A interfaces, the ground-state geometry structures were optimized using DFT/B3LYP/6-31G(d), and the absorption spectra were calculated using TD-DFT/CAM-B3LYP/6-31G(d) based on the optimized ground-state geometry structures of D/A interfaces. Partial density of states (PDOS) were visualized with GaussSum software (Cambridge, UK) [42]. The charge density difference (CDD) plots of D/A interfaces were visualized with Multiwfn 3.3.9 package [43]. For the dimer system, the difference configurations should produce the different couple strength, and the direction of stacking has a certain influence on the estimation of charge mobility. Experimentally, PDBT-T1:IDIC and PTFBDT-BZS:IDIC blended films processed with 0.25% DIO exhibit higher *J_SC_* and FF, finally higher PCE, showing that the vertical π-π stacking is favorable to charge transport between anode and cathode of solar cells [31]. Meanwhile, stacking mode of π-π stacking for the polymers was reported in Refs. [31,32]. Considering the above mention, the face-to-face stacking was used to optimize the dimer configurations and to further estimate the electron transfer and charge transport. To evaluate electronic coupling matrix of D/A interfaces, the Generalized Mulliken-Hush (GMH) model [44] and the finite field method [45,46] on the excitation energy of the D/A interfaces were employed. All calculations were performed with Gaussian 09 program package [47].

The TD-DFT calculations provide the singlet excited states $|S_{n}\rangle$ (*n* defined as number *n* state) represented by vectors $C_{n,ai}^{CI}$ based on configurations of unoccupied and occupied molecular orbital’s *a* and *i*, respectively. The molecular orbitals are given by linear combinations of atomic orbitals (*LCAO*) *μ* and with coefficients $c_{a\mu }^{LCAO}$ and $c_{i\nu }^{LCAO}$. For characterizing the observations of excited states, we use the two matrixes: [48]

(1) $$Q_{\mu \nu }^{\left(n\right)}=\frac{1}{\sqrt{2}}\sum_{\begin{array}{l} a\in unocc\\ i\in occ \end{array}}C_{n,ai}^{CI}\left(c_{a\mu }^{LCAO}c_{i\nu }^{LCAO}+c_{i\mu }^{LCAO}c_{a\nu }^{LCAO}\right)$$

(2) $$P_{\mu \nu }^{\left(n\right)}=\frac{i}{\sqrt{2}}\sum_{\begin{array}{l} a\in unocc\\ i\in occ \end{array}}C_{n,ai}^{CI}\left(c_{a\mu }^{LCAO}c_{i\nu }^{LCAO}-c_{i\mu }^{LCAO}c_{a\nu }^{LCAO}\right)$$

which are (anti) symmetric for exchange of the atomic orbitals and normalized as:

(3) $$\sum_{\mu ,\nu }|Q_{\mu \nu }^{\left(n\right)}{|}^{2}=\sum_{\mu ,\nu }|P_{\mu \nu }^{\left(n\right)}{|}^{2}=1$$

Within the framework of the collective electron oscillator (CEO) model, the excited state $|S_{n}\rangle$ is described by a coordinate $Q_{\mu \nu }^{\left(n\right)}\cos(\omega_{n}t)$ and a momentum $P_{\mu \nu }^{\left(n\right)}\sin(\omega_{n}t)$, both oscillating with the transition frequency $\omega_{n}$. The matrixes $Q_{\mu \nu }^{\left(n\right)}$ and $P_{\mu \nu }^{\left(n\right)}$ can be employed to visual characterization of the excited states.

In real space, the oscillating CEO coordinate and momentum are expressed by the spatial correlation functions: [48]

(4) $$Q_{n}(r,\;r^{\prime };\;t)=\sum_{\mu \nu }\phi_{\mu }^{AO}(r)Q_{\mu \nu }^{\left(n\right)}\phi_{\nu }^{AO}(r^{\prime })\cos(\omega_{n})$$

(5) $$P_{n}(r,\;r^{\prime };\;t)=\sum_{\mu \nu }\phi_{\mu }^{AO}(r)P_{\mu \nu }^{\left(n\right)}\phi_{\nu }^{AO}(r^{\prime })\sin(\omega_{n})$$

The diagonal slice for $r=r^{\prime }$ results in:

(6) $$Q_{n}(r,r;t)=\sqrt{2}\rho_{n0}(r)\cos(\omega t)$$

(7) $$P_{n}(r,r;t)=0$$

Thereby, the charge density difference (CDD) can be given by: [49]

(8) $$\Delta \rho_{nn}(r)=2i\sum_{\mu ,\nu ,\kappa }\phi_{\mu }^{AO}(r)Q_{\kappa \nu }^{\left(n\right)}P_{\kappa \nu }^{\left(n\right)}\phi_{\nu }^{AO}(r)$$

It represents the difference of electron distribution between the excited state $|S_{n}\rangle$ and the ground state $|S_{0}\rangle$. Therefore, the charge density difference can tell the difference of electron density of the excited state $|S_{n}\rangle$ and the ground state $|S_{0}\rangle$.

## 3. Results and Discussion

### 3.1. Geometric Structures and Electronic Properties of IDIC, IDTBR And Polymers

**Figure 2:**
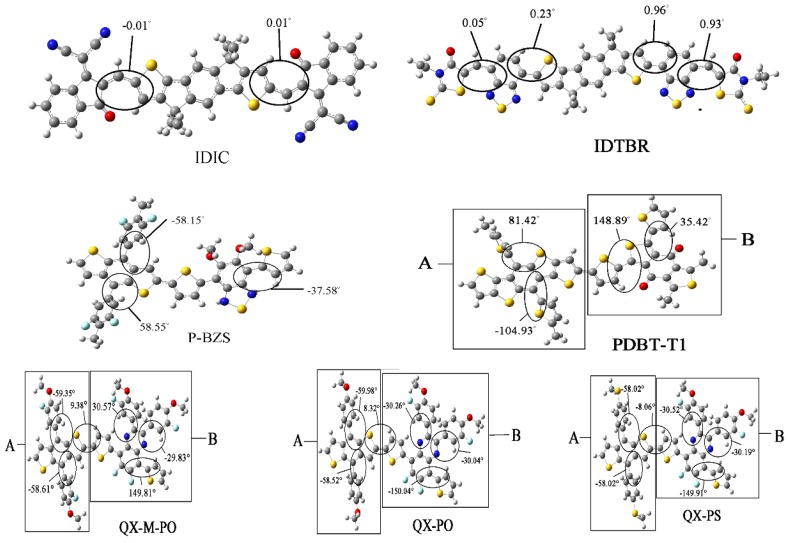
The optimized ground-state geometry structures, important dihedral angels, and definitions of molecular fragments of two NFAs, IDIC and IDTBR, and five kinds of polymers.

**Figure 3:**
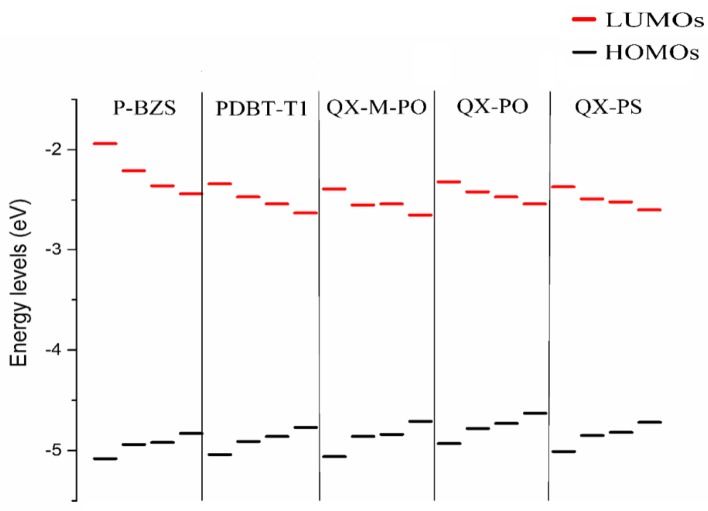
Energy levels of all oligomers (*n* = 1–3 and *n* = ∞), where the black line and red line stand HOMO and LUMO, respectively.

**Figure 4:**
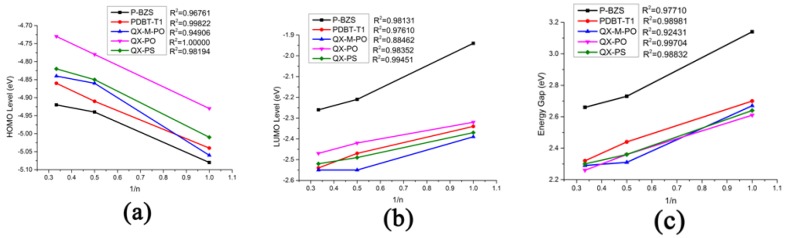
The relationship between energy levels of polymers and the reciprocal of conjugated chain (1/*n*): (**a**) HOMO; (**b**) LUMO; and (**c**) energy gap.

The energy levels in frontier molecular orbitals (FMOs) and the energy gaps for the polymer are directly related to the open circuit voltage, optical properties and charge dissociations of solar cells [50,51]. By using DFT/B3LYP/6-31G(d), simulated results include the obtained levels of HOMO and LUMO and the energy gaps (Δ_H-L_) of the five kinds of the polymers (*n* = 1, 2, 3), as displayed in Table 1. Besides, the FMOs levels of the NFAs (IDIC and IDTBR) are listed in Table 2. The optimized ground-state geometry structures, important dihedral angels and definitions of molecular fragments of IDIC, IDTBR and the five kinds of polymers are presented in Figure 2. Energy levels of all oligomers (*n* = 1–3 and *n* = ∞) are displayed in Figure 3. Figure 4 depicts the relationship between the HOMO energy levels, LUMO energy levels and energy gap (Δ_H-L_) of polymers and the reciprocal of conjugated unit (1/*n*).

As shown in Figure 2, the two NFAs (IDIC and IDTBR) exhibit a good flatness. From the data about the main dihedral angles that influence the planar of two molecules, it can be found that these dihedral angles are very small, almost close to zero, which can be considered as the excellent co-planarity for the two molecules. While for the five types of the polymers, the important dihedral angles of main chain are relatively large, displaying an obvious distortion under the backbones of the five polymers.

In Table 1 and Figure 3, it was found that, with the increase of conjugated chains, the HOMOs levels of polymers are all increased, and LUMOs levels are all decreased. Therefore, the energy gaps of the five polymers have a certain decreasing trend. When the conjugated unit *n* = 3, the HOMO levels of the five polymers are in the following order: P-BZS < PDBT-T1 < QX-M-PO < QX-PS < QX-PO; and the LUMO levels are in the order: P-BZS > QX-PO > QX-PS > PDBT-T1 > QX-M-PO. For the three polymers (QX-PO, QX-PS, and QX-M-PO, with the same unit 5,8-Bis(5-thiophen-2-yl)-6,7-difluoro-2,3-bis(4-ethylhexyloxy-1-meta-fluorophenyl)quinoxaline (ffQx)), the introduction of F group in 2,6-bis(trimethyltin)-4,8-bis(4-ethylhexyloxy-1-phenyl)-benzo (1,2-b:4,5-b0)-dithiophene for QX-M-PO make the LUMO lower than the QX-PS with the substitution of S atom in QX-PO. The LUMO levels of above polymers are all higher than those of NFAs, which is helpful for the charge transfer in the interface.

As shown in Figure 4, the HOMO energy levels, LUMO energy levels, and the energy gaps of the five polymers have good linear relationships with the reciprocal of conjugated chain (1/*n*) (all adjusted *R*-square (*R*^2^) are close or equal to 1, as shown in Figure 4). Due to the good linear relationship, we can predict the HOMO level, LUMO level and ∆_H-L_ when the conjugated unit *n*→∞ by using linear fitting method theoretically. As shown in Table 1, when the *n*→∞, the HOMO energy levels of P-BZS, PDBT-T1, QX-M-PO, QX-PO, and QX-PS are −4.83, −4.77, −4.71, −4.63, and −4.72 eV, respectively. The LUMO energy levels are −2.44, −2.63, −2.65, −2.54, and −2.60 eV, respectively. The energy gaps are 2.39, 2.14, 2.06, 2.09, and 2.12 eV, respectively. Among the five polymers, P-BZS have the deepest HOMO level and the highest LUMO level. In calculation, the energy gaps of five polymers have following order (*n* = ∞): P-BZS > PDBT-T1 > QX-PS > QX-PO > QX-M-PO. The HOMO levels of IDIC and IDTBR are (see Table 2) −5.76 and −5.21 eV, respectively, while their LUMO levels are −3.51 and −3.31 eV, which are all higher than that of PCMB (−3.85 eV [18]). Bumjoon J. Kim et al. found that the higher performance was mainly attributed to the enhanced *V_OC_* value owing to the higher-lying unoccupied molecular orbital compared with fullerene derivative [18].

**Figure 5:**
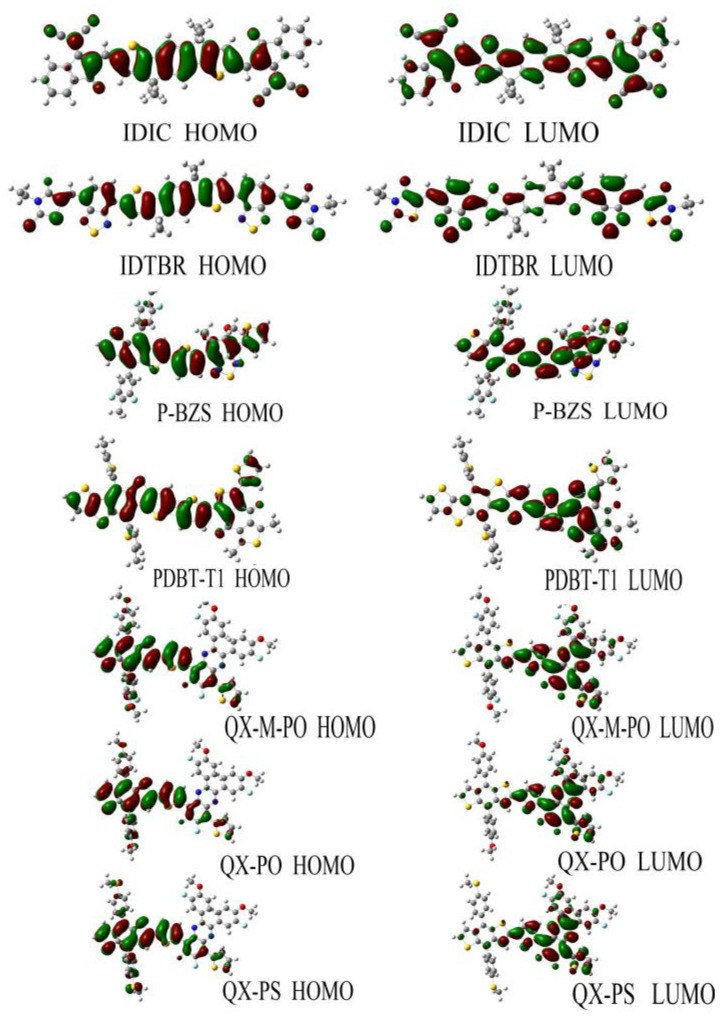
The frontier molecular orbital plots of the NFAs (IDIC, IDTBR) and the five kinds of polymers (*n* = 1), where the red color represents electrons and green represents holes, and “A” and “B” fragments are defined in Figure 2.

Figure 5 shows the FMOs of IDIC, IDTBR and the five kinds of polymers (*n* = 1). For IDIC, the electronic clouds HOMO and LUMO are evenly distributed over the molecular backbone. Compared to HOMO, LUMO has more distribution on the benzene ring at both ends of the molecule, indicating that the benzene ring at both ends has better electron withdrawing power than the rest part of the molecule. For IDTBR and P-BZS, HOMO and LUMO electron clouds are uniformly distributed on the molecular backbone, indicating that there are no visible electron donor moieties and electron acceptor moieties on IDTBR and the P-BZS. For the sake of clear understanding the distribution of electron, we define fragments for PDBT-T1, QX-M-PO, QX-PO, and QX-PS, and the definitions of the fragment are shown in Figure 2. It can be seen in Figure 5, for these four polymers, the HOMO has more distribution in their “A” fragments, and LUMO in fragment “B” is more obvious. Note that for these four kinds of polymers, their “A” fragments are the electron donor moieties of molecules, while their “B” fragments are the electron acceptor portions of molecules.

Open circuit voltage (*V_OC_*) is one of the key factors governing the performance of a photovoltaic cell [35]. For the D/A interfaces, the *V_OC_* can be estimated by following equation theoretically [52]:

(9) $$V_{OC}=\frac{1}{e}(|E_{HOMO}(D)-E_{LUMO}(A)|)-\Delta V$$

where *e* represents the element electron; *E_HOMO_*(*D*) and *E_LUMO_*(*A*) represent the HOMO level of donor and the LUMO level of acceptor, respectively; and Δ*V* is an empirical factor, which can be assumed to be a value of 0.5 V [51]. The *V_OC_* of the polymer/IDTBR devices and the polymer/IDIC devices are listed in Table 3. For the polymers/IDIC devices, the *V_OC_* values are in this order: P-BZS/IDIC > QX-M-PO/IDIC > PDBT-T1/IDIC > QX-PS/IDIC > QX-PO/IDIC. For the polymers/IDTBR devices, the *V_OC_* are in order of: P-BZS/IDTBR > QX-M-PO/IDTBR > PDBT-T1/IDTBR > QX-PS/IDTBR > QX-PO/IDTBR. Because P-BZS has the deepest HOMO energy level among the five polymers, the P-BZS-based device has the largest *V_OC_* value for the polymers/IDIC devices and polymers/IDTBR, i.e., their *V_OC_* reach 1.07 and 1.27 eV, respectively. Note that, for each kind of polymer, the *V_OC_* value of the polymer/IDTBR device is higher than that of the polymers/IDIC device due to the higher LUMO levels of IDTBR, which means that, for the same donor, the higher LUMO level of acceptor can produce a larger *V_OC_*.

### 3.2. IP and EA of the Nfas And The Five Polymers

**Figure 6:**
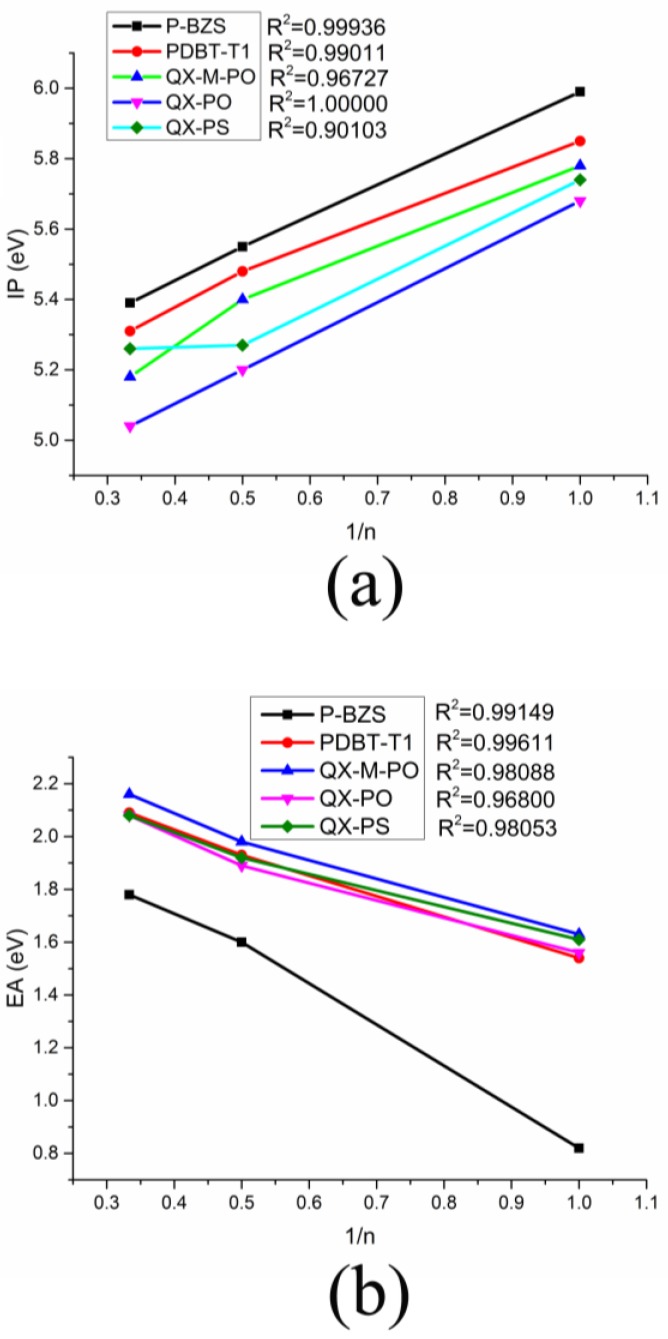
The relationship between the IPs and EAs of the polymers and the reciprocal of the conjugated chain (1/*n*), where (**a**,**b**) corresponding to IP and EA, respectively.

Ionization potentials (IPs) and electron affinities (EAs) can be used to estimate the transport barrier of holes and electrons in the organic solar cells. In general, the lower IP means a lower barrier of the holes injection, and the higher EA indicates the electrons injection become easier [53,54]. The results of IPs and EAs of IDIC, IDTBR and five polymers are listed in Table 4. Figure 6 shows the relationship between the IPs (EAs) of the polymers with the reciprocal of the conjugated chain (1/*n*), which displays a good linear relationship.

It can found in Table 4 that, for the five polymers, when the conjugated chain of polymers increases, the IPs decreases and the EAs increases gradually, which means the fact that both hole and electron injection of polymers are facilitated along with the increase of molecular conjugated chains. As shown in Figure 6, we can predict the theoretical IPs and EAs when the *n*→∞ through the linear fitting method due to the good linear relationships between the IPs (EAs) of the polymers with the reciprocal of the conjugated chain (1/*n*) (All Adjusted *R*-square (*R*^2^) are close even equal to 1, as shown in Figure 6). When the *n*→∞, the IPs of P-BZS, PDBT-T1, QX-M-PO, QX-PO, and QX-PS are calculated to be 5.10, 5.06, 4.92, 4.72, and 4.95 eV, respectively, while their EAs are 2.30, 2.35, 2.40, 2.28, and 2.29 eV, respectively. The IPs and EAs of the five kinds of polymers are very close. This shows that the five polymers should have similar electron injection and hole injection capabilities. For IDIC and IDTBR, their IPs are 6.59 and 5.94 eV, and their EAs are 2.73 and 2.60 eV, respectively. Apparently, IDIC has higher EA than IDTBR, meaning that IDIC has better electron injection ability. The IDIC and IDTBR have higher IP and higher EA than the five polymers, which manifests that IDIC and IDTBR have better electron injection abilities than those of polymers, and the five polymers have better hole injection abilities than those of the NFAs.

### 3.3. The Reorganization Energies of Polymers and NFAs

The reorganization energy can be used to estimate the charge transfer characteristic of organic material, and the lower reorganization energy proclaims the faster charge transport [54]. The internal reorganization energies λ*_h_* (hole reorganization energy) and λ*_e_* (electron reorganization energy) can be expressed by following formulas [55]:

(10) $$\lambda_{h}=(E_{0}^{+}-E_{+})+(E_{+}^{0}-E_{0})$$

(11) $$\lambda_{e}=(E_{0}^{-}-E_{-})+(E_{-}^{0}-E_{0})$$

where $E_{0}^{+}$ ($E_{0}^{-}$) represents the energies of the cation (anion) calculated with the optimized structure of the neutral molecular; *E*_+_ (*E*_−_) is the energy of the cation (anion) calculated with the optimized cation (anion) structure; $E_{0}^{+}$ ($E_{0}^{-}$) is the energy of the neutral molecular calculated at the cationic (anionic) state; and *E*_0_ is the energy of the neutral molecule at the ground state. The calculated reorganization energies of IDIC, IDTBR and all the oligomers (*n* = 1–3) are shown in Table 5. In Table 5, we can discover that, along with the increase of conjugated chains, both λ*_h_* and λ*_e_* of the five polymers are reduced gradually, which manifests that, with the increasing conjugated chains of polymers, the electrons transport and holes transport are both enhanced. When the conjugated chain *n* = 3, it can be seen that the differences in holes reorganization energies of the five polymers is very small, which can predict the closed level of electron transport rate and hole transport rate for the five polymers. For IDIC and IDTBR, their λ*_h_* are 0.15 and 0.16 eV, respectively, while their λ*_e_* are 0.21 and 0.17 eV, respectively. As can be seen, IDTBR has better electron transport capability than IDIC.

### 3.4. Absorption Spectra of the Five Polymers and of IDIC, IDTBR

**Figure 7:**
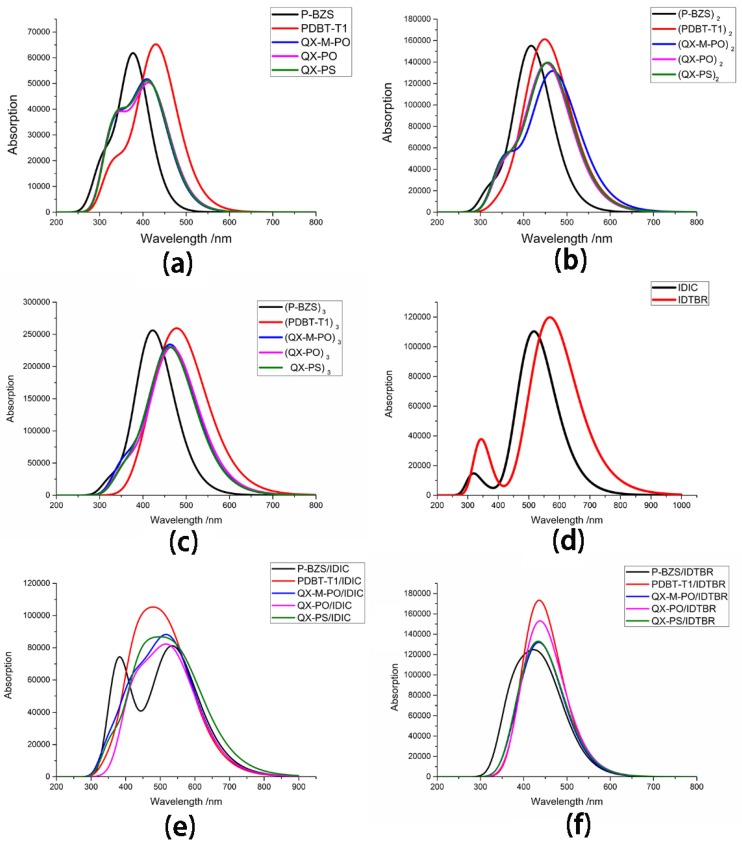
Absorption spectra of all the oligomers for: (**a**) *n* = 1; (**b**) *n* = 2; and (**c**) *n* = 3; (**d**) absorption spectra of NFAs IDIC and IDTBR; (**e**) absorption spectra of D/A interfaces polymer/IDTBR; and (**f**) absorption spectra of D/A interfaces polymer/IDIC.

The polymers, as an electron donor in the solar cell devices, should have a strong and wide optical absorption range, which will match with the solar spectrum well [35]. Based on the optimized ground state structures of IDIC, IDTBR and the five kinds of polymers, their simulated optical absorption spectra were calculated with the TD-DFT/CAM-B3LYP/6-31G (d). The absorption peaks and corresponding oscillator strengths of oligomers (*n* = 1–3) are listed in Table 6, and the transition energies and oscillators strength for IDIC and IDTBR are listed in Table 7. The absorption spectra of the five polymers for *n* = 1–3 and absorption spectra of IDIC and IDTBR are presented in Figure 7. Besides, the transition energies and oscillator strengths of the six excited states for the polymers (*n* = 1) are listed in Table S1 in Supplementary Materials.

In Table 6, it was found that for, each kind of polymer, with the increase of the conjugated chain, the absorption peak of each polymer is increased, and the corresponding oscillator strength is the same. Moreover, for all oligomers (*n* = 1–3), the excited state S1 is originated from the electron transition from HOMO to LUMO. We obtained the first absorption peaks of tripolymers (P-BZS, PDBT-T1, QX-M-PO, QX-PO, and QX-PS) are 432.67, 492.90, 474.19, 478.58, and 473.32 nm, respectively, while their corresponding oscillator strengths are 4.3297, 5.1319, 4.1316, 4.0453, and 4.2384, respectively. The absorption peaks of tripolymers are in following sequence: PDBT-T1 > QX-PO > QX-M-PO > QX-PS > P-BZS; and their corresponding oscillator strengths are in this order: PDBT-T1 > P-BZS > QX-PS > QX-M-PO > QX-PO. In Figure 7, we can confirm that for each kind of polymer, the absorption peak is increased with the increase of the conjugated chain. As shown in Figure 7, the PDBT-T1 have better optical absorption than the P-BZS, for *n* = 1–3; the three polymers QX-M-PO, QX-PO and QX-PS have similar optical absorption. In addition, with the increase of conjugated chain of polymer, absorption ranges of the five polymers have obviously bathochromic-shifted and broadened range, which shows that, with the increase of conjugated chain of polymers, the optical absorptions of five polymers all become better. When the conjugated unit *n* = 3, for P-BZS, the absorption range is about from 275 to 650 nm; for PDBT-T1, the absorption range is about 325 to 750 nm; and, for QX-M-PO, QX-PO and QX-PS, the absorption ranges are about 300 to 700 nm. Among the five polymers for *n* = 1–3, it was found that the PDBT-T1 have the widest optical absorption range, the maximum absorption peak and the maximum oscillator strength in the five polymers, meaning that PDBT-T1 has the best optical absorption properties in the five polymers. Comparison of the polymers QX-M-PO, QX-PO, and QX-PS indicates that these three polymers have similar absorption peaks, oscillator strengths and absorption ranges, meaning that the molecular design by the introduction of F group or the substitution of S atom has no obvious influence on the absorption peaks.

The transition energies and oscillators of IDIC and IDTBR are listed in Table 7, including the transition information of the first six excited states for IDIC and IDTBR, and their absorption spectra are presented in Figure 7. As shown, for IDIC and IDTBR, the absorption peaks of S1 are at 516.84 and 569.58 nm, respectively; the corresponding oscillator strengths are 2.7244 and 2.9442, respectively; and the transition orbitals are both from HOMO to LUMO. Comparing with IDIC, the absorption peak of IDTBR is red-shifted about 52.74 nm, and the transition energy of IDTBR is reduced by 0.22 eV. As can be seen in Figure 7, the absorption range of IDIC is about from 250 to 800 nm, and that of TDTBR is about from 250 to 950 nm. The absorption spectra of IDTBR are significantly red-shifted compared with that of IDIC, and the absorption range of IDTBR is wider than that of IDIC. It is clearly seen that IDTBR have better optical absorption capability than that of IDIC. Comparing with the five polymers, both two NFAs has the greater first absorption peaks and bigger oscillator strengths in comparison with the five polymers.

### 3.5. The Hole/Electron Mobility Rate and Hole/Electron Mobility of the Five Polymers and of IDIC and IDTBR

There exist the two models to describe the mechanism of charge transport: the coherent band model and the thermally activated hopping model [56,57]. At the very low temperature, a band-like model describes the transport mechanisms in the well-ordered organic materials. In this case, it is found that both electron and hole transports fall into the coherent band-like regime [58]. At room temperature, the charge transfer in organic semiconductor with weak intermolecular interactions is generally regarded to happen through the thermally activated hopping model [59,60]. In this case, the charge carriers are localized on a single molecule, jumping from one molecule to the adjacent molecule, and the Marcus theory is a widely used method to estimate the charge hoping rate, which can be expressed as [61,62,63]:

(12) $$K=\frac{V^{2}}{\hbar }{\left(\frac{\pi }{\lambda k_{B}T}\right)}^{\frac{1}{2}}\exp\left(-\frac{\lambda }{4k_{B}T}\right)$$

In this equation, *K* is the rate of charge transfer for electrons and holes (*K_e_* and *K_h_*, respectively), *V* is the charge transfer integral, *ħ* is Planck’s constant, *k_B_* is the Boltzmann constant, *T* is room temperature (setting *T* = 300 K in our work), and λ is the reorganization energy of charge transfer process. Here, it is found that the charge transfer rate depends on two key parameters (*V* and λ).

For the electrons and holes, the charge transfer integrals are written as *V_e_* and *V_h_*, respectively. We use following equations to measure *V_e_* and *V_h_* [64,65]:

(13) $$V_{e}=\frac{E_{L+1}-E_{L}}{2}$$

(14) $$V_{h}=\frac{E_{H}-E_{H-1}}{2}$$

where *E_L+_*_1_, *E_L_*, *E_H_* and *E_H_*_−1_ represent the energy levels of LUMO + 1, LUMO, HOMO and HOMO-1, respectively. Analogously, for the electrons and holes, the reorganization energies are λ*_e_* and λ*_h_*, given in Formulas (10) and (11).

According to the Einstein relation, the drift mobility of hopping *μ* is usually evaluated from the Einstein–Smoluchowski equation: [58,66]

(15) $$\mu =\frac{e}{k_{B}T}D$$

where *e* is the electron charge, *k_B_* is the Boltzmann constant, *T* is room temperature, and *D* is the diffusion coefficient. The diffusion coefficient *D* can be evaluated from the hopping rates as: [56,60]

(16) $$D=\underset{t\to \infty }{\lim}\frac{1}{2d}\frac{\langle x{\left(t\right)}^{2}\rangle }{t}\approx \frac{1}{2d}\sum_{m}r_{m}^{2}k_{m}p_{m}$$

where *k_m_* is the hopping rate due to the charge carrier to the *m*th neighbor, and *r_m_* is the distant to neighbor *m*, and *p_m_* is the relative probability for charge carrier to a particular *m*th neighbor. In addition, when considering only one neighbor, the diffusion constant along a single molecular dimer is simply defined as: [67]

(17) $$D=\frac{1}{2}Kr^{2}$$

where *K* and *r* are the rate of charge mobility and intermolecular distance for the dimer, respectively. At room temperature, the drift mobility of hopping *μ* can be expressed as [65,68]:

(18) $$\mu =\frac{er^{2}}{2k_{B}T}K$$

The important parameters linked with the drift mobility of the NFAs (IDIC and IDTBR) and the five polymers are listed in Table 8, and in calculations the dimer structures of IDIC, IDTBR and the five polymers are face-to-face dimer structure, which can be seen in Figure S1 in Supplementary Materials. As shown in Table 8, for IDIC and IDTBR, the electron transfer rates *K_e_* are 1.27 × 10^10^ and 1.25 × 10^14^ s^−1^, respectively. Obviously, IDTBR has much faster electron transfer rate than IDIC. For the five polymers, P-BZS, PDBT-T1, QX-M-PO, QX-PO, and QX-PS, their *K_h_* are 1.44 × 10^13^, 4.17 × 10^12^, 3.37 × 10^12^, 3.97 × 10^11^ and 2.34 × 10^12^ s^−1^ respectively. By employing Equation (18), we can obtain that the electron mobilities *μ_e_* of IDIC and IDTBR are 7.36 × 10^−4^ and 6.3043 cm^2^/(V·s), respectively; for the five polymers, their *μ_h_* are 0.6080, 0.1460, 0.1420, 0.1730, and 0.1020 cm^2^/(V·s), respectively. IDITBR has much greater electron mobility than IDIC, which because IDTBR has much larger *K_e_* than IDIC. 

### 3.6. The Ground-State Properties of D/A Interfaces

**Figure 8:**
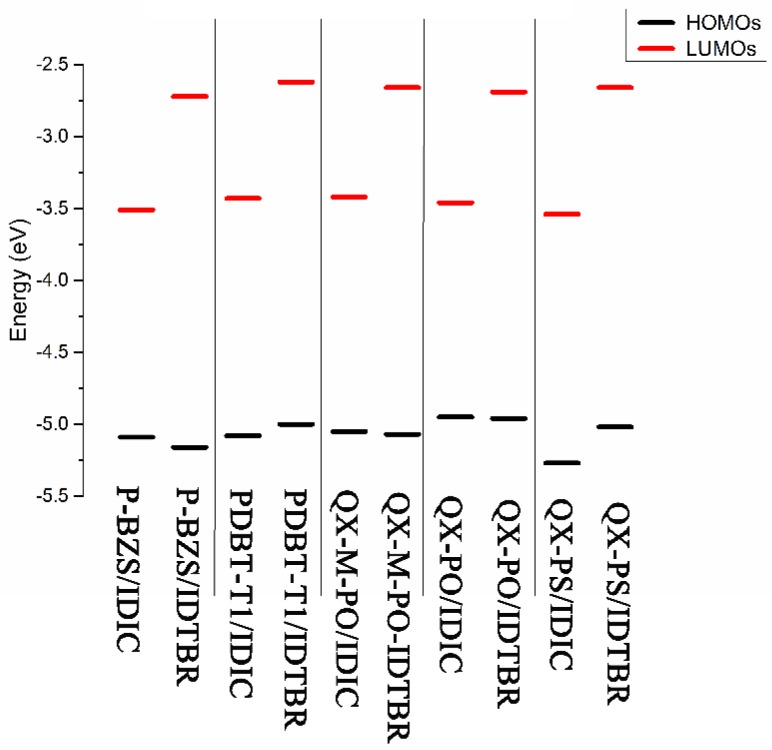
The FMOs energy levels of the five polymer/IDIC interfaces and the five polymer/IDTBR interfaces, where black line represents HOMO level and red line represents LUMO level.

**Figure 9:**
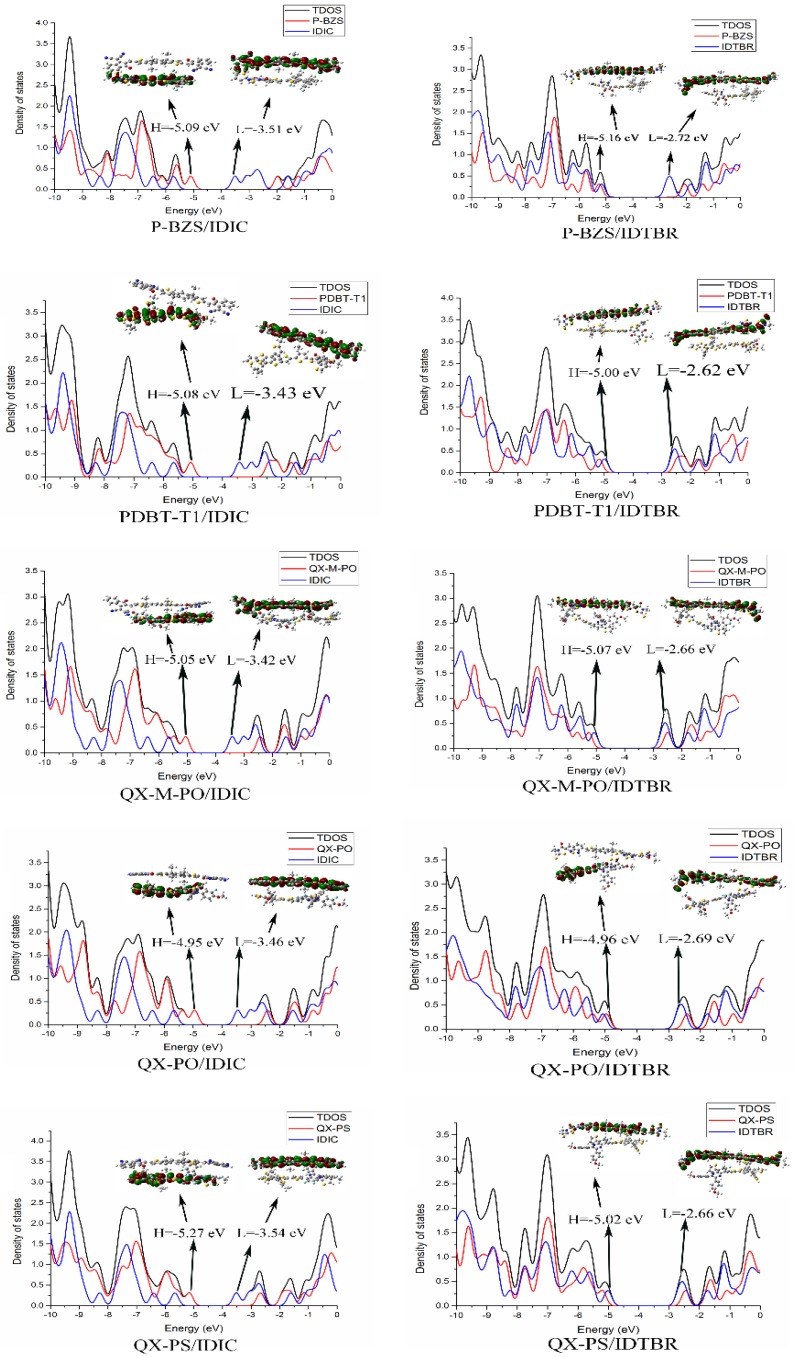
The partial density of states (PDOS) plots and frontier molecular orbital plots of ten D/A interfaces (For all D/A interfaces, the NFAs are on the top, and the polymers are underneath).

The ground-state geometry structures of the ten D/A interfaces have been optimized by using DFT/B3LYP/6-31G(d). The energy levels and energy gaps for the ten D/A interfaces were shown in Table S2 in Supplementary Materials. Based on the optimized ground state geometries of D/A interfaces, the partial density of states (PDOS) were calculated. The FMOs energy level of the five polymer/IDIC interfaces and the five polymer/IDTBR interfaces are depicted in Figure 8. Besides, Figure 9 presents the PDOS plots, FMOs plots and FMO levels of the ten D/A interfaces.

In Table S2 and Figure 8, we can find that for the polymers/IDIC interfaces and polymers/IDTBR interface; their HOMO energy levels are not very different; and the energy levels of LUMO vary greatly. The LUMO energy levels of the D/A interfaces show a trend of: polymer/IDTBR > polymer/IDIC. Compared with polymer/IDIC interfaces, the LUMO of P-BZS/IDTBR, PDBT-T1/IDTBR, QX-M-PO/IDTBR, QX-PO/IDTBR, and QX-PS/IDTBR interfaces are increased about 0.79, 0.81, 0.76, 0.77 and 0.88 eV, respectively. 

In Figure 9, with the PDOS, we can find the percentage contribution of some groups to each molecular orbital [5]. As shown in Figure 9, for the five polymer/IDIC interfaces, the contribution of HOMO mainly comes from the polymers, and the LUMO is mainly contributed by IDIC. The corresponding FMOs plots can prove this point. For the five classes of polymer/IDIC interfaces, one can see that the electronic clouds of HOMO are located on the polymer, and LUMO’s electronic cloud are located on IDIC, which proves that in polymer/IDIC, the polymers dominate HOMO levels, and IDIC dominates LUMO levels. For the five kinds of polymer/IDTBR interfaces, the HOMOs of the other four types of IDITBR-based interfaces are mainly contributed by IDTBR except for QX-PO; the contribution of LUMO are all from IDTBR. For the QX-PO/IDTBR, its HOMO is located at the QX-PO, and LUMO is from IDTBR, which is supported by the FMOs plots. Except for the QX-PO/IDIC, the HOMO and LUMO of the remaining four kinds of IDTBR-based interfaces are localized on the IDTBR, while QX-PO/IDTBR’s HOMO is localized on QX-PO, and LUMO distributes on IDTBR.

### 3.7. Ips and Eas for Ten D/A Interfaces

**Figure 10:**
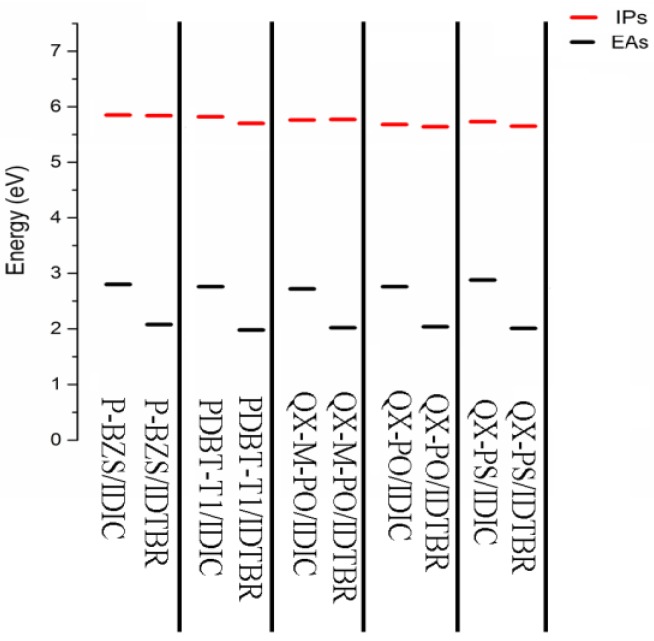
The IPs (EAs) of five polymer/IDIC interfaces and five polymer/IDTBR interfaces, where black line represents EA and red line represents IP.

The calculated Ips and Eas of the ten D/A interfaces are listed in Table 9. The Ips/EA of the five Polymer/IDIC interfaces and the five Polymer/IDTBR interfaces are shown in Figure 10. Analyzing Table 9 and Figure 10, we can find that for the IDIC-based Interfaces and the IDTBR-based interfaces, the differences of IP are very small; however, the differences of EA are obvious. Eas are showing trends of Polymer/IDIC > Polymer/IDTBR. It is obvious that the electron injection abilities of IDIC-based interfaces are better than those of IDTBR-based interfaces. compared with IDTBR-based interfaces, the Eas of IDIC-based interfaces are increased about 0.72, 0.78, 0.70, 0.72 and 0.87 eV, respectively.

### 3.8. Optical Absorption for Ten D/A Interfaces

Light is absorbed by the organic layer, and electron–hole pairs are generated; as mentioned, the absorption characteristics of the organic layer should match the solar spectrum as closely as possible. Based on the optimized ground-state geometries of the ten D/A interfaces, the electronic transitions in optical absorption were calculated with TD-DFT/CAM-B3LYP/6-31G (d). The electronic transitions of the first six excited state for the polymer/IDIC interfaces are listed in Table 10, and the electronic transitions of the first six excited state for polymer/IDTBR interfaces are listed in Table 11. The absorption spectra of polymer/IDIC interfaces and polymer/IDTBR interfaces are presented in Figure 7.

In Table 10, we can see that the first absorption peaks of P-BZS/IDIC, PDBT-T1/IDIC, QX-M-PO/IDIC, QX-PO/IDIC, and QX-PS/IDIC are at 535.23, 527.60, 532.08, 534.00, and 555.21 nm, respectively; and their corresponding oscillator strengths are 1.9897, 1.9649, 1.8682, 1.8247, and 1.6883, respectively. In addition to the QX-PO/IDIC, the transition orbitals of S1 for the polymer/IDIC are from HOMO-1 to LUMO, and the transition orbitals of S1 for QX-PO/IDIC is from HOMO-2 to LUMO. The transition orbitals of S2 for the five polymer/IDIC interfaces are from HOMO to LUMO. The maximum absorption peaks are in the order: QX-PS/IDIC > P-BZS/IDIC > QX-PO/IDIC ≈ QX-M-PO/IDIC > PDBT-T1/IDIC; and the corresponding oscillator strengths are in the order: P-BZS/IDIC > PDBT-T1/IDIC > QX-M-PO/IDIC > QX-PO/IDIC > QX-PS/IDIC. For the two manufactured D/A interfaces, it is obvious that P-BZS/IDIC have a better optical absorption property than that of PDBT-T1/IDIC, which coincides with the experimental results [31]. Among the five polymer/IDIC interfaces, P-BZS/IDIC has the second largest absorption peak and the greatest oscillator strength, and QX-PS/IDIC has the largest absorption peak, meaning that these two D/A interfaces have the best optical absorption properties among the five D/A interfaces. Two new designed D/A interfaces: QX-M-PO/IDIC and QX-PO/IDIC show the optical performance close to the synthesized D/A interface P-BZS/IDIC; and the newly designed QX-PS/IDIC even shows a better optical absorption performance than P-BZS/IDIC. It shows that the five IDIC-based interfaces exhibit excellent optical absorption properties. Comparing with the polymers (*n* = 1), it is noted that for, P-BZS, PDBT-T1, QX-M-PO, QX-PO, and QX-PS, when the D/A interfaces with IDIC was constructed, the first absorption peaks of them have bathochromic shifted about 156.21, 92.38, 115.85, 114.75, and 137.42 nm, respectively; and their corresponding oscillator strengths have increased about 0.5034, 0.4023, 0.6847, 0.6582, and 0.5136, respectively. Furthermore, as shown in Figure 7, the five kinds of IDIC-based interfaces have strong and wide absorption ranges. For P-BZS/IDIC, PDBT-T1/IDIC, QX-M-PO/IDIC, and QX-PO/IDIC, the absorption ranges are all about from 300 to 850 nm, while, for QX-PS/IDIC, the absorption range is about from 300 to 900 nm. Comparing with the five polymers, we can find that their corresponding polymer/IDIC interfaces have wider and more red-shifted absorption ranges. When the polymers were matched with IDIC to form D/A interfaces, the absorption peaks of them have obviously red shifted, and their corresponding oscillator strengths have obvert increases.

For polymer/IDTBR interfaces, the maximum absorption peaks of P-BZS/IDTBR, PDBT-T1/IDTBR, QX-M-PO/IDTBR, QX-PO/IDTBR, and QX-PS/IDTBR are 446.80, 452.85, 448.32, 452.44, and 453.96 nm; and their oscillators strengths are 2.4542, 1.1034, 2.5731, 2.3682, and 2.2361, respectively. In addition to the QX-PO/IDTBR, the transition orbitals of S1 for polymer/IDTBR are from HOMO to LUMO, and the transition orbitals of QX-PO/IDTBR are from HOMO-1 to LUMO. As presented in Figure 7, the absorption ranges of the five polymer/IDTBR interfaces are all about from 300 to 650 nm. Comparing with five polymers (*n* = 1), the maximum absorption peaks of their corresponding polymer/IDTBR interfaces make red-shifted about 67.78, 20.63, 32.09, 33.19, and 36.17 nm, respectively; and the maximum oscillator strengths of their corresponding polymer/IDTBR interfaces were raised by 0.9679, 1.6971, 1.3896, 1.2071, and 1.0614, respectively. Moreover, compared with the five kinds of polymers, the optical absorption ranges of the five systems (polymer/IDTBR) are obviously bathochromic shifted. Furthermore, when comparing IDIC-based interfaces with IDTBR-based interfaces, it is found that for the five kinds of polymers, their corresponding IDIC-based interfaces have larger absorption peaks than that of IDTBR-based interfaces, except for the smaller oscillator strengths. It is clearly demonstrated that the sunlight absorption ranges of IDIC-based interfaces is much wider and more bathochromic-shifted than those of IDTBR-based interfaces. Therefore, comparing the original five polymers, their corresponding polymer/non-fullerene interfaces have better sunlight absorption properties; comparing IDIC-based interfaces with IDTBR-based interfaces, the IDIC-based interfaces have much better optical absorption performances than the IDTBR-based interfaces.

**Figure 11:**
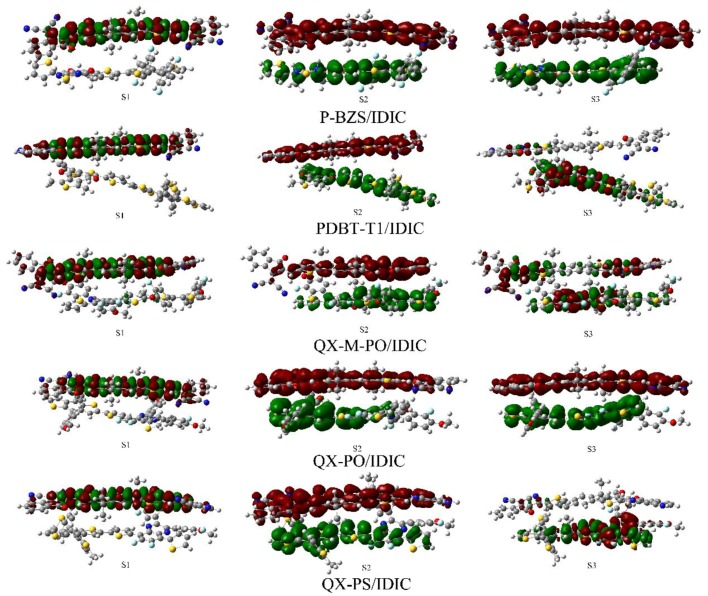
Charge difference densities (CDD) plots of polymer/IDIC interfaces (S1–S3), where red represents electrons and green represents holes.

**Figure 12:**
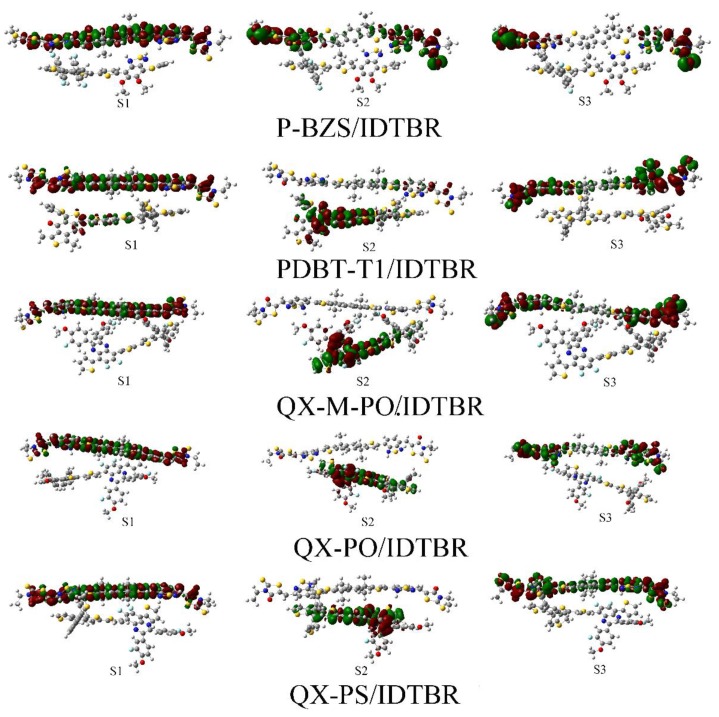
Charge difference densities (CDD) plots of polymer/IDTBR interfaces (S1–S3), where red represents electrons and green represents holes.

The quantum chemistry methods coupled with CDD methods have been used to study the structure and charge transfer character of organic molecules under photo-excitation [69,70,71]. It is hoped that effective charge separation can be occurred under the heterojunction. Therefore, we did the calculation about CDD plots for the first six excited states of the ten D/A interfaces. The CDD plots of the polymer/IDIC interfaces are presented in Figure 11 (excited states S1–S3) and Figure S2 in Supplementary Materials (S4–S6), and the CDD plots of polymer/IDTBR are drawn in Figure 12 (S1–S3) and Figure S3 in Supplementary Materials (S4–S6). In Figure 11, for the S1 of the five polymer/IDIC interfaces, the electrons and holes are both located at IDIC. It is worth noting that charge transfer can be found in the second and third state. For example, for the S2 of the five polymer/IDIC interfaces, the electrons are only located at IDIC, and the holes are only located at polymers, which means the electrons is transferred from the polymers to the acceptor IDIC. The electron transfer type is view as an intermolecular charge transfer [45,72]. Under the excited state S2, the electrons are separated effectively for the five polymer/IDIC interfaces. In the first six excited states of the IDIC-based interfaces, the efficient electronic separation states are S2, S3, and S5 for P-BZS/IDIC; S2 and S5 for the PDBT-T1/IDIC; S2, S4, and S6 for the QX-M-PO/IDIC; S2, S3, and S6 for QX-PO/IDIC; and S2, S4, S5, and S6 for QX-PS/IDIC. As shown in Figure 11, the effective charge separation can take place at each D/A interface. Obviously, in the first six excited states of the five IDIC-based interfaces, QX-PS/IDIC has more effective charge separated state than the other four interfaces; PDBT-T1/IDIC compared to other interfaces has the least effective charge separated state; and P-BZS/IDIC, QX-M-PO/IDIC, and QX-PO/IDIC are the same as the charge separation state.

For the S1 of the five polymer/IDTBR interfaces, the electrons and holes are both distribute on the acceptor IDTBR, which is similar to that IDIC-based interfaces, indicating that the S1 of IDTBR-based interfaces are all occur at IDTBR. For both IDIC-based interfaces and IDTBR-based interfaces, the S1 of them respectively occur at two NFAs. Except for P-BZS/IDTBR, for S2 of four polymer/IDTBR interfaces, the electrons and holes are both located in the polymers, suggesting that the electrons transfer takes place on the polymer. For S2 of P-BZS/IDTBR, the electrons and holes are both distribute on IDTBR, indicating that the electron transfer happen at IDTBR. In the first six excited states, there are no efficient electronic separation happened in the five polymer/IDTBR interfaces. That explains why, for each kind of polymer, the absorption peaks of their corresponding IDTBR-based interfaces are blue-shifted compared to those of their corresponding IDIC-based polymers. From the CDD plots of D/A interfaces, we can observe the electron transfer processes and characteristics of each excited state, and to observe the roles of the polymers and the NFAs in each excited state.

### 3.9. Exciton Binding Energies of Ten D/A Interfaces

When the organic layer absorbs sunlight, the dominant species produced is an exciton, which is an electron/hole pair bound by Coulombic attraction rather than the free charge carriers [73]. After the exciton is formed, it will transport to the D/A interface, and then the exciton will be separated under interface [74]. The main steps are [75]: (i) charge transfer from the excited singlet state S1 to the CT state; and (ii) escape of the electron–hole pair forming the CT state from their mutual Coulombic potential well. To dissociate exciton to charges successfully, large exciton binding energy has to be overcame. The exciton binding energy is one of the key parameters of organic solar cell devices, and it is directly related to the charge separation in organic solar cells. A system with small *E_b_* often possesses high charge separation efficiency, which is beneficial for photovoltaic applications [75]. Theoretically, exciton binding energy is estimated by the following expression [48,76,77,78]:

(19) $$E_{b}=IP-EA-E_{opt}$$

where *IP* and *EA* are the ionization potential and electron affinity, respectively; and *E_opt_* is the optical band gap D/A interface. The exciton binding energy of D/A interfaces should be in the range of 0 to 1.5 eV [79,80,81,82]. Optical band gap energy (*E_opt_*) and exciton binding energy (*E_b_*) of the ten D/A interfaces are listed in Table 9. As shown, for P-BZS/IDIC, PDBT-T1/IDIC, QX-M-PO/IDIC, QX-PO/IDIC, and QX-PS/IDIC, the *E_b_* are 0.73, 0.80, 0.71, 0.60, 0.62 eV, respectively; and for P-BZS/IDTBR, PDBT-T1/IDTBR, QX-M-PO/IDTBR, QX-PO/IDTBR, and QX-PS/IDTBR, the *E_b_* are 0.98, 0.98, 0.98, 0.87, and 0.91 eV, respectively. For each kind of polymer, the IDIC-based interfaces have the less *E_b_* than that the IDTBR-based interfaces (that is to say: P-BZS/IDIC < P-BZS/IDTBR, PDBT-T1/IDIC < PDBT-T1/IDTBR and so on). It suggests that the IDIC-based interfaces need to overcome smaller exciton binding energies in comparison with the IDTBR-based interfaces; that is, the exciton is easier to be dissociated into free charge in the IDIC-based interfaces.

### 3.10. The Rates of Charge Separation and the Rates of Charge Recombination of Polymer/IDIC Interfaces

The IDIC-based interfaces have better electron injection abilities, better optical absorption properties and smaller exciton binding energies than IDTBR-based interfaces; besides, efficient charge separation have occurred in IDIC-based interfaces; we calculated the charge separation rates and charge recombination rates of polymer/IDIC interfaces. In the semi-classical limit of Marcus theory, the charge-transfer rate can be expressed as [80,83]:

(20) $$K=\sqrt{\frac{4\pi^{3}}{h^{2}\lambda k_{B}T}}|V_{DA}{|}^{2}\exp\left(-\frac{\left(\Delta G+\lambda \right)}{4\lambda k_{B}T}\right)$$

where *V_DA_* is the charge transfer integrals between the initial and final states, *λ* is the reorganization energy of D/A interface, which usually can be expressed as the sum of the intramolecular reorganization energy and the outer reorganization energy. Δ*G* is the vibration of the Gibbs free energy during the reaction, which can be expressed as Δ*G*_CS_ and Δ*G*_CR_ in the charge separation process and charge recombination processes, respectively. *k_B_* is the Boltzmann’s constant, *h* is the Planck’s constant, and *T* is the temperature; usually, we use room temperature *T* = 300 K.

The charge transfer integrals *V_DA_* is to be evaluated in a diabatic description where the initial and final states do not interact. In this case, *V_DA_* can be estimated from the quantities given by CI (configuration interaction) calculations performed on the interacting donor/acceptor pair by using the generalized Mulliken-Hush (GMH) formalism, which refers to an optical process between the two states. *V_DA_* is then expressed as: [84]

(21) $$V_{DA}=\frac{\mu_{tr}\Delta E}{\sqrt{{\left(\Delta \mu \right)}^{2}+4{\left(\mu_{tr}\right)}^{2}}}$$

where Δ*E*, Δ*μ*, and *μ_tr_* correspond to the energy difference, the dipole moment difference, and the transition dipole moment between the initial and final states, respectively. This formalism is particularly attractive since it covers the photo-induced CT processes and allows the inclusion of electron correlation in the description of the relevant states [85]. The dipole moment difference in Equation (21) can be calculated by using Hellmann–Feynman theorem. The dipole moments difference of excited states is estimated by using finite field method. The transition energy dependent on the static electric field *F* can be expressed as: [46,86,87]

(22) $$E_{ext}(F)=E_{ext}(0)-\Delta \mu F-\frac{1}{2}\Delta \alpha F^{2}$$

where $E_{ext}(0)=\Delta E$ is the excitation energy of the lowest intermolecular charge transfer excited state at zero field, and Δα is the change in polarizability. Calculated charge transfer integrals for the polymer/IDIC interfaces are listed in Table 12. The charge transfer integrals *V_DA_* of IDIC-based interfaces are 0.2177, 0.2721, 0.8816, 0.1061, and 1.034 eV, respectively. The *V_DA_* of the IDIC-based interfaces are following this sequence: QX-PS/IDIC > QX-M-PO/IDIC > PDBT-T1/IDIC > P-BZS/IDIC > QX-PO/IDIC.

The reorganization energy is a key parameter to calculate charge transfer rate, and the intramolecular reorganization energy refers to the change in the energy of the system due to the relaxation of the geometric structure when the electron gain/loss, or the electronic state changes [79]; the outer reorganization energy is due to the electronic and nuclear polarization/relaxation of the surrounding medium [55,88]. The overall intramolecular reorganization energy consists of two terms [85]:

(23) $$\lambda_{in}=\lambda_{in}(A)+\lambda_{in}(D)$$

(24) $$\lambda_{in}(D)=E(D)-E(D^{+})$$

(25) $$\lambda_{in}(A)=E(A^{-})-E(A)$$

where *E*(*D*) and *E*(*D*^+^) are the energies of the radical cation *D*^+^ at the neutral geometry and optimal cation geometry, respectively; and *E*(*A*^−^) and *E*(*A*), accordingly, represent the energies of the neutral acceptor *A* at the anionic geometry and the energies of neutral acceptor at optimal ground-state geometry, respectively. By employing quantum chemical theory, it is a hard work to accurately calculate outer reorganization energy *λ_s_* in the D/A interface, especially for our new designed D/A interfaces. In our work, the value of *λ_s_* is viewed as a constant equal to 0.3 eV [28,89].

For charge recombination process, the Δ*G_CR_* can be estimated with [90]:

(26) $$\Delta G_{CR}=E_{IP}(D)-E_{EA}(A)$$

where *E_IP_*(*D*) represents the ionization potential of the donor, and *E_EA_*(*A*) is the electron affinity of the acceptor. As an approximation, the Gibbs free energy change of charge separation process is estimated from the Rehm-Weller equation: [91]

(27) $$\Delta G_{CS}=-\Delta G_{CR}-E_{S1}-E_{b}$$

where *E_S_*_1_ and *E_b_* are the energy of lowest excited state of free-base donor and exciton binding energy of D/A interface, respectively. 

The important parameters associated with the charge-transfer rate of D/A interfaces are listed in Table 13. As shown, all the D/A interfaces have close reorganization energies. For P-BZS/IDIC, PDBT-T1/IDIC, QX-M-PO/IDIC, QX-PO/IDIC, and QX-PS/IDIC, their Δ*G_CS_* are −0.74, −0.55, −0.64, −0.60, and −0.58 eV, respectively; and their Δ*G_CR_* are −3.26, −3.12, −3.05, −2.95, and −3.01 eV, respectively. The charge separation rates *K*_CS_ of P-BZS/IDIC, PDBT-T1/IDIC, QX-M-PO/IDIC, QX-PO/IDIC, and QX-PS/IDIC are 2.29 × 10^13^, 3.20 × 10^13^, 6.54 × 10^14^, 2.87 × 10^12^, and 3.60 × 10^14^ s^−1^, respectively. We cannot figure out the K_CR_ of QX-PO/IDIC, while the *K*_CR_ of P-BZS/IDIC, PDBT-T1/IDIC, QX-M-PO/IDIC, and QX-PS/IDIC are 2.14 × 10^5^, 4.20 × 10^2^, 1.51 × 10^5^, and 3.68 × 10^6^, respectively. The results show that, among the five kinds of D/A interfaces, QX-M-PO/IDIC and QX-PS/IDIC have the best charge separation rates, which are larger than the *K*_CS_ of the two synthesized D/A interfaces (P-BZS/IDIC and PDBT-T1/IDIC). QX-PO/IDIC has the smallest charge separation rate. This indicates that in the five kinds of the polymer, polymers QX-M-PO and QX-PS combined with IDIC to form the corresponding D/A interfaces are most favorable for the charges separation. In addition to the QX-PO/IDIC, the *K*_CS_/*K*_CR_ for P-BZS/IDIC, PDBT-T1/IDIC, QX-M-PO/IDIC, QX-PS/IDIC are 1.07 × 10^8^, 7.69 × 10^9^, 4.33 × 10^9^, 9.78 × 10^8^, respectively. As the *K*_CS_/*K*_CR_ is shown, for the four D/A interfaces, their charge separation rates are much larger than their charge recombination rates, which indicates that the electronic separation can be achieved effectively in these four polymer/IDIC interfaces. As for QX-PS, because it has a very large charge separation rate, we can assume that the charge separation is also effective at this D/A interface. In terms of the D/A interfaces, the greater charge separation rate and the smaller charge recombination rate can promote the *J_SC_* of the solar cell device [37]. For polymer/IDIC interfaces, they all have larger *K*_CS_ and smaller *K*_CR_; thus, we can think that such IDIC-based interfaces will have appreciable *J_SC_*, furthermore, producing an appreciable PCEs. 

## 4. Conclusions

In this work, two non-Fullerene Acceptors (NFAs: IDIC and IDTBR) and five polymers were selected and investigated by DFT and TD-DFT. Based on the optimized ground-sated structures of NFAs and the five polymers, we studied the ten D/A interfaces, which include the two synthesized D/A interfaces (P-BZS/IDIC and PDBT-T1/IDIC) and eight newly designed D/A interfaces, by employing quantum-chemical method and Marcus semi-classical model. The results demonstrated that: (a) As the substitution of fullerene derivatives, the LUMO of the two NFAS are all higher than that of PCMB, which can enhance the value of *V_OC_* > (b) Among the five polymers, PDBT-T1 has largest absorption peak in the visible region, and polymers QX-M-PO and QX-PO have similar optical response, indicating that the introduction of F group in 2,6-bis(trimethyltin)-4,8-bis(4-ethylhexyloxy-1-phenyl)-benzo[1,2-b:4,5-b0]-dithiophene for QX-M-PO has not influence on the absorption spectra. For the construction of D/A interfaces, the absorption peak of D/A make the red-shifted compared with the single polymer, which promote the sufficient utilization of sunlight for the dimer system. (c) Using the same polymers coupled with IDTBR or IDIC to compare the performance of the two NFAs, the smaller exciton binding of the IDIC-based system indicates the charge separation should take place more easily, and charge different density provides the visualized evidence (where the electron (red color) was moved from the polymer to IDIC for the S2). Meanwhile, the Marcus semi-classical model demonstrated that the charge separation rate polymer/IDIC interfaces is about six orders of magnitude higher than the charge recombination rate. Finally, we infer that IDIC-based interfaces have better performance in the utility of BHJ solar cell than IDTBR-based interfaces. We hope that our investigations in this work can further provide theoretical guidance for optimizing OSCs acceptor materials and achieve a breakthrough on the dilemmas of existing polymer solar cells. 

## Figures and Tables

**Table 1:** Energy levels of the highest occupied molecular orbital (HOMO (H)), the lowest unoccupied molecular orbital (LUMO (L)) (eV) and the energy gap Δ_H-L_ (eV) for all oligomers.

		P-BZS	PDBT-T1	QX-M-PO	QX-PO	QX-PS
*n* = 1	H	−5.08	−5.04	−5.06	−4.93	−5.01
	L	−1.94	−2.34	−2.39	−2.32	−2.37
	Δ_H-L_	3.14	2.70	2.67	2.61	2.64
*n* = 2	H	−4.94	−4.91	−4.86	−4.78	−4.85
	L	−2.21	−2.47	−2.55	−2.42	−2.49
	Δ_H-L_	2.73	2.44	2.31	2.36	2.36
*n* = 3	H	−4.92	−4.86	−4.84	−4.73	−4.82
	L	−2.26	−2.54	−2.55	−2.47	−2.52
	Δ_H-L_	2.66	2.32	2.29	2.26	2.30
*n* = ∞	H	−4.83	−4.77	−4.71	−4.63	−4.72
	L	−2.44	−2.63	−2.65	−2.54	−2.60
	Δ_H-L_	2.39	2.14	2.06	2.09	2.12

**Table 2:** Energy levels of HOMO (eV), LUMO (eV) and energy gap Δ_H-L_ (eV) for Non-fullerene acceptors (NFAs) IDIC, IDTBR.

	IDIC	IDTBR
H	−5.76	−5.21
L	−3.51	−3.31
Δ_H-L_	2.25	1.90

**Table 3:** The open voltage *V_OC_* (eV) of polymer/IDIC and polymer/IDTBR devices; where the conjugated unit of polymer *n* = 1.

	Acceptors	IDIC	IDTBR
Polymers			
P-BZS		1.07	1.27
PDBT-T1		1.03	1.23
QX-M-PO		1.05	1.25
QX-PO		0.92	1.22
QX-PS		1.00	1.30

**Table 4:** The ionization potentials IP (eV) and electron affinities EA (eV) of the five polymers and of the NFAs IDIC, IDTBR.

Molecules	IP				EA			
	*n* = 1	*n* = 2	*n* = 3	*n* = ∞	*n* = 1	*n* = 2	*n* = 3	*n* = ∞
P-BZS	5.99	5.55	5.39	5.10	0.82	1.60	1.78	2.30
PDBT-T1	5.85	5.48	5.31	5.06	1.54	1.93	2.09	2.35
QX-M-PO	5.78	5.40	5.18	4.92	1.63	1.98	2.16	2.40
QX-PO	5.68	5.20	5.04	4.72	1.56	1.86	2.08	2.28
QX-PS	5.74	5.27	5.26	4.95	1.61	1.92	2.08	2.29
IDIC	6.59				2.73			
IDTBR	5.94				2.60			

**Table 5:** The reorganization energies of oligomers (*n* = 1–3) and the NFAs.

Molecules	λ*_h_* (eV)			λ*_e_* (eV)		
	*n* = 1	*n* = 2	*n* = 3	*n* = 1	*n* = 2	*n* = 3
P-BZS	0.60	0.19	0.15	0.36	0.22	0.17
PDBT-T1	0.24	0.15	0.10	0.33	0.21	0.14
QX-M-PO	0.31	0.22	0.19	0.36	0.18	0.15
QX-PO	0.29	0.24	0.19	0.39	0.18	0.13
QX-PS	0.27	0.23	0.16	0.42	0.18	0.13
IDIC	0.15			0.21		
IDTBR	0.16			0.17		

**Table 6:** The absorption peaks and corresponding oscillator strengths oligomers (*n* = 1–3).

Molecule	State	*E* (eV)	Absorption peak λ (nm)	Contribution MOs	Strength *f*
P-BZS					
*n* = 1	S1	3.27	379.02	H→L (0.66639)	1.4863
*n* = 2	S1	2.93	423.18	H→L (0.58778)	3.4568
*n* = 3	S1	2.87	432.67	H→L (0.51443)	4.3297
PDBT-T1					
*n* = 1	S1	2.87	432.22	H→L (0.64008)	1.5626
*n* = 2	S1	2.64	469.57	H→L (0.54709)	2.2491
*n* = 3	S1	2.52	492.90	H→L (0.49314)	5.1319
QX-M-PO					
*n* = 1	S1	2.98	416.23	H→L (0.59187)	1.1835
*n* = 2	S1	2.61	474.92	H→L (0.56328)	2.7947
*n* = 3	S1	2.61	474.19	H→L (0.51806)	4.1361
QX-PO					
*n* = 1	S1	2.96	419.25	H→L (0.57410)	1.1665
*n* = 2	S1	2.69	461.33	H→L (0.55638)	2.9821
*n* = 3	S1	2.59	478.58	H→L (0.51439)	4.0453
QX-PS					
*n* = 1	S1	2.97	417.79	H→L (0.57592)	1.1747
*n* = 2	S1	2.68	462.33	H→L (0.56559)	3.0549
*n* = 3	S1	2.61	473.32	H→L (0.51369)	4.2384

**Table 7:** Transition energies and oscillators strengths for the NFAs (IDIC, IDTBR).

Molecule	State	*E* (eV)	Absorption peak λ (nm)	Contribution MOs	Strength *f*
IDIC					
	S1	2.40	516.84	H→L (0.65169)	2.7244
	S2	3.01	411.41	H→L + 1 (0.57513)	0.0000
	S3	3.46	358.86	H→L + 2 (0.57087)	0.0939
	S4	3.52	352.25	H→L + 3 (0.50408)	0.0000
	S5	3.56	348.57	H-7→L + 2 (0.38485)	0.0000
	S6	3.56	348.54	H-8→L + 2 (0.38494)	0.0000
IDTBR					
	S1	2.18	569.58	H→L (0.62354)	2.9442
	S2	2.52	491.55	H→L + 1 (0.56766)	0.0280
	S3	3.08	402.52	H-4→L + 2 (0.56766)	0.0001
	S4	3.08	402.11	H-3→L + 3 (0.37743)	0.0001
	S5	3.27	378.78	H-1→L (0.46724)	0.0129
	S6	3.30	375.64	H-2→L (0.46724)	0.0104

**Table 8:** The key parameters related to drift mobility of NFAs (IDIC and IDTBR) and the five polymers: *V_e_*/*V_h_* (eV), λ*_e_*/λ*_h_* (eV), *r* (Å), *K_e_*/*K_h_* (s^−1^) and *μ_e_*/*μ_h_* (cm^2^/(V·s)).

	***V_e_***	**λ*_e_***	***k_e_***	***r***	***μ_e_***
IDIC	0.00163	0.21	1.27 × 10^10^	5.47	7.36 × 10^−4^
IDTBR	0.12626	0.17	1.25 × 10^14^	5.11	6.3043
	***V_h_***	***λ_h_***	***k_h_***	***r***	***μ_h_***
P-BZS	0.04875	0.19	1.44 × 10^13^	4.67	0.6080
PDBT-T1	0.02027	0.15	4.17 × 10^12^	4.26	0.1460
QX-M-PO	0.02816	0.22	3.37 × 10^12^	4.67	0.1420
QX-PO	0.01088	0.24	3.97 × 10^11^	4.75	0.1730
QX-PS	0.02490	0.23	2.34 × 10^12^	4.75	0.1020

**Table 9:** The IPs, EAs, optical band gap energies (*E*_opt_) and exciton binding energies (*E*_b_) of D/A interfaces.

D/A Interfaces	IP (eV)	EA (eV)	*E*_opt_ (eV)	*E*_b_ (eV)
P-BZS/IDIC	5.85	2.80	2.32	0.73
PDBT-T1/IDIC	5.82	2.76	2.35	0.80
QX-M-PO/IDIC	5.76	2.72	2.33	0.71
QX-PO/IDIC	5.68	2.76	2.32	0.60
QX-PS/IDIC	5.73	2.88	2.23	0.62
P-BZS/IDTBR	5.84	2.08	2.78	0.98
PDBT-T1/IDTBR	5.70	1.98	2.74	0.98
QX-M-PO/IDTBR	5.77	2.02	2.77	0.98
QX-PO/IDTBR	5.65	2.04	2.74	0.87
QX-PS/IDTBR	5.65	2.01	2.73	0.91

**Table 10:** Transition energies and oscillator strengths for D/A interfaces polymer/IDIC.

Interfaces	*E* (eV)	λ (nm)	Contribution MOs	Strength *f*
P-BZS/IDIC				
S1	2.32	535.23	H-1→L (0.64571)	1.9897
S2	2.49	497.32	H→L (0.68663)	0.0059
S3	2.86	433.45	H→L + 1 (0.50061)	0.0007
S4	2.94	422.04	H-1→L + 1 (0.56022)	0.0223
S5	3.19	388.35	H-3→L (0.60443)	0.0089
S6	3.23	383.57	H→L + 3 (0.58721)	1.3310
PDBT-T1/IDIC				
S1	2.35	527.60	H-1→L (0.64572)	1.9649
S2	2.67	464.94	H→L (0.67118)	0.0142
S3	2.85	434.89	H→L + 3 (0.59640)	1.7541
S4	2.96	418.71	H-1→L + 1 (0.54964)	0.1524
S5	3.10	399.40	H→L + 1 (0.56169)	0.0030
S6	3.36	369.24	H→L + 5 (0.36596)	0.1091
QX-M-PO/IDIC				
S1	2.33	532.08	H-1→L (0.58892)	1.8682
S2	2.45	505.90	H→L (0.58745)	0.1400
S3	2.90	426.36	H→L + 3 (0.42461)	0.8127
S4	2.93	423.46	H-2→L (0.47518)	0.0052
S5	2.96	418.36	H-1→L + 2 (0.39677)	0.4739
S6	3.16	392.25	H→L + 1 (0.38327)	0.0012
QX-PO/IDIC				
S1	2.32	534.00	H-2→L (0.63872)	1.8247
S2	2.34	527.66	H→L (0.60302)	0.0004
S3	2.78	446.38	H-1→L (0.52415)	0.0002
S4	2.89	428.49	H→L + 3 (0.52044)	1.0973
S5	2.95	420.88	H-2→L + 1 (0.52727)	0.2355
S6	3.10	399.94	H→L + 1 (0.42268)	0.0008
QX-PS/IDIC				
S1	2.23	555.21	H-1→L (0.63726)	1.6883
S2	2.48	500.40	H→L (0.65873)	0.0233
S3	2.77	447.31	H→L + 2 (0.56768)	1.6181
S4	2.88	430.67	H-2→L (0.40210)	0.0287
S5	2.90	427.38	H-1→L + 1 (0.47503)	0.0114
S6	3.19	388.04	H→L + 1 (0.43135)	0.0004

**Table 11:** Transition energies and oscillator strengths for D/A interfaces polymer/IDTBR.

Interfaces	*E* (eV)	λ (nm)	Contribution MOs	Strength *f*
P-BZS/IDTBR				
S1	2.76	446.80	H→L (0.56871)	2.4542
S2	3.12	396.71	H-2→L (0.29578)	0.0864
S3	3.14	393.98	H-9→L (0.33442)	0.0164
S4	3.18	389.59	H-10→L (0.31034)	0.0599
S5	3.27	378.70	H-1→L + 2 (0.56327)	1.2629
S6	3.32	373.10	H-1→L + 2 (0.30340)	0.6293
PDBT-T1/IDTBR				
S1	2.73	452.85	H→L (0.53040)	1.1034
S2	2.87	430.94	H-1→L + 2 (0.55550)	3.2597
S3	3.10	399.81	H-2→L (0.39061)	0.0078
S4	3.14	394.40	H-8→L + 6 (0.22903)	0.0082
S5	3.16	391.27	H-3→L (0.25714)	0.0070
S6	3.25	381.18	H-3→L (0.31279)	0.0033
QX-M-PO/IDTBR				
S1	2.76	448.32	H→L (0.56150)	2.5731
S2	3.10	398.82	H-1→L + 2 (0.49594)	1.1560
S3	3.12	396.56	H-2→L + 2 (0.30762)	0.0270
S4	3.15	392.71	H-4→L (0.24075)	0.0052
S5	3.20	387.09	H→L + 1 (0.27965)	0.0153
S6	3.29	376.77	H-4→L (0.45729)	0.0345
QX-PO/IDTBR				
S1	2.74	452.44	H-1→L (0.56657)	2.3682
S2	2.96	418.23	H→L + 2 (0.51852)	1.6954
S3	3.12	397.29	H-3→L (0.32364)	0.0056
S4	3.15	392.60	H-4→L (0.32696)	0.0025
S5	3.21	385.85	H-1→L + 1 (0.29098)	0.0122
S6	3.28	377.86	H-4→L (0.31635)	0.0156
QX-PS/IDTBR				
S1	2.73	453.96	H→L (0.55344)	2.2361
S2	3.06	405.17	H-1→L + 1 (0.47892)	1.5389
S3	3.10	399.61	H-3→L (0.36520)	0.0589
S4	3.15	392.68	H-9→L + 6 (0.25236)	0.0040
S5	3.20	387.43	H-5→L (0.24538)	0.0154
S6	3.29	376.08	H-5→L (0.28417)	0.0650

**Table 12:** Calculated charge transfer integrals for the polymer/IDIC interfaces.

D/A Interfaces	States	Δ*μ* (a.u)	*μ_tr_* (a.u)	Δ*E* (eV)	*V_DA_* (eV)
P-BZS/IDIC	S2	−3.511	0.3117	2.4931	0.2177
PDBT-T1/IDIC	S2	4.457	0.4659	2.6667	0.2721
QX-M-PO/IDIC	S2	2.945	1.5271	2.4507	0.8816
QX-PO/IDIC	S2	−1.927	0.0883	2.3497	0.1061
QX-PS/IDIC	S2	−0.817	0.6197	2.4777	1.034

**Table 13:** Dynamic parameters of the polymers/IDIC interfaces: inner reorganization energy λ*_in_* (eV), outer reorganization energy λ*_s_* (eV), free enthalpy of the reaction Δ*G* (eV), the rates of exciton-separation *k*_CS_ (s^−1^), and charge-recombination *k*_CR_ (s^−1^).

	P-BZS/IDIC	PDBT-T1/IDIC	QX-M-PO/IDIC	QX-PO/IDIC	QX-PS/IDIC
*λ_in_*	1.15	0.92	0.95	1.05	0.99
*λ_s_*	0.30	0.30	0.30	0.30	0.30
*λ*	1.45	1.22	1.25	1.35	1.29
Δ*G_CS_*	−0.74	−0.55	−0.64	−0.60	−0.58
Δ*G_CR_*	−3.26	−3.12	−3.05	−2.95	−3.01
*V*	0.2177	0.2721	0.8816	0.1061	1.034
*k*_CS_	2.29 × 10^13^	3.20 × 10^13^	6.54 × 10^14^	2.87 × 10^12^	3.60 × 10^14^
*k*_CR_	2.14 × 10^5^	4.20 × 10^2^	1.51 × 10^5^	--	3.68 × 10^6^

## Supplementary Materials

The following are available online at www.mdpi.com/2073-4360/9/12/692/s1.

## Abbreviations

OSC	Organic solar cell
OPV	Photovoltaics
BHJ	Bulk-heterojunction
PSC	Polymer solar cell
All-PSCs	All-polymer organic solar cells
PCE	Power conversion efficiencies
NFAs	Non-fullerene acceptors
DFT	Density functional theory
TD-DFT	Time-dependent density functional theory
PDOS	Partial density of states
CDD	Charge density difference
FMOs	Frontier molecular orbitals
HOMO	The highest occupied molecular orbital
LUMO	The lowest unoccupied molecular orbital
IP	Ionization potential
EA	Electron affinity
