# Supplementary material for: Non-Fullerene Acceptor-Based Solar Cells: From Structural Design to Interface Charge Separation and Charge Transport
Source: Polymers (Basel). 2017 Dec 8;9(12):692. doi: 10.3390/polym9120692 (PMC6418710; doi:10.3390/polym9120692)
Supplement: Supplementary file 1 [file polymers-09-00692-s001.pdf]

**Supporting Materials**

---

Non-Fullerene Acceptor-Based Solar Cells: From structural design to Interface Charge Separation and Charge Transport

Qungui Wang[a],Yuanzuo Li[a,*], Peng Song[b], Runzhou Shu [a], Fengcai Ma[b], Yanhui Yang[c,*]

[a]*College of Science, Northeast Forestry University, Harbin 150040, Heilongjiang, China, Email：yzli@nefu.edu.cn;*

[b]*Department of Physics, Liaoning University, Shenyang 110036, Liaoning, China;*

[c]*School of Chemical and Biomedical Engineering, Nanyang Technological University,Singapore;Email:YHYang@ntu.edu.sg.*

**Table S1.Transition energies and oscillators for polymers for n=1.**

| molecule | state | E (eV) | Absorption peak λ(nm) | Contribution MOs | Strength *f* |
|---|---|---|---|---|---|
| P-BZS | S1 | 3.27 | 379.02 | H→L (0.66639) | 1.4863 |
| | S2 | 3.89 | 319.15 | H-1→L (0.58321) | 0.3679 |
| | S3 | 3.99 | 310.98 | H-2→L (0.55825) | 0.0352 |
| | S4 | 4.14 | 299.37 | H→L+1 (0.29482) | 0.0379 |
| | S5 | 4.18 | 296.12 | H→L+1 (0.38820) | 0.1495 |
| | S6 | 4.45 | 278.19 | H→L+2 (0.42496) | 0.0399 |
| PDBT-T1 | S1 | 2.87 | 432.22 | H→L (0.64008) | 1.5626 |
| | S2 | 3.36 | 368.95 | H→L+1 (0.40539) | 0.1691 |
| | S3 | 3.54 | 349.90 | H→L+2 (0.31763) | 0.1216 |
| | S4 | 3.67 | 336.43 | H-1→L (0.27323) | 0.0087 |
| | S5 | 3.76 | 329.13 | H-1→L (0.28423) | 0.1897 |
| | S6 | 3.87 | 320.20 | H-2→L (0.35574) | 0.1479 |
| QX-M-PO | S1 | 2.98 | 416.23 | H→L (0.59187) | 1.1835 |
| | S2 | 3.55 | 349.24 | H-2→L (0.55776) | 0.5059 |
| | S3 | 3.62 | 342.05 | H→L+1 (0.43208) | 0.1296 |
| | S4 | 3.86 | 320.90 | H-1→L+1 (0.44586) | 0.3408 |
| | S5 | 3.87 | 320.01 | H-8→L (0.24759) | 0.0060 |
| | S6 | 4.03 | 307.12 | H-3→L (0.36064) | 0.0402 |
| QX-PO | S1 | 2.95 | 419.25 | H→L (0.57410) | 1.1665 |
| | S2 | 3.55 | 348.96 | H-2→L (0.50658) | 0.5498 |
| | S3 | 3.60 | 343.81 | H→L+1 (0.36701) | 0.0475 |
| | S4 | 3.84 | 322.66 | H-1→L+1 (0.44080) | 0.3852 |
| | S5 | 3.87 | 319.80 | H-14→L (0.24393) | 0.0084 |
| | S6 | 4.01 | 308.99 | H-3→L (0.36862) | 0.0487 |
| QX-PS | S1 | 2.96 | 417.79 | H→L (0.57592) | 1.1747 |
| | S2 | 3.55 | 349.23 | H-2→L (0.53626) | 0.5458 |
| | S3 | 3.61 | 343.45 | H→L+1 (0.40308) | 0.0877 |
| | S4 | 3.84 | 322.16 | H-1→L+1 (0.44722) | 0.3727 |
| | S5 | 3.87 | 320.11 | H-8→L (0.25663) | 0.0075 |
| | S6 | 4.02 | 308.23 | H→L+2 (0.30622) | 0.0255 |

**Table S2. Energy levels of HOMO (H) (eV), LUMO (L) (eV) and energy gap ΔH-L (eV) for ten D/A interfaces.**

| Interfaces | H | L | $\Delta_{H-L}$ |
|---|---|---|---|
| P-BZS/IDIC | -5.09 | -3.51 | 1.58 |
| PDBT-T1/IDIC | -5.08 | -3.43 | 1.65 |
| QX-M-PO/IDIC | -5.05 | -3.42 | 1.63 |
| QX-PO/IDIC | -4.95 | -3.46 | 1.49 |
| QX-PS/IDIC | -5.27 | -3.54 | 1.73 |
| P-BZS/IDTBR | -5.16 | -2.72 | 2.44 |
| PDBT-T1/IDTBR | -5.00 | -2.62 | 2.38 |
| QX-M-PO/IDTBR | -5.07 | -2.66 | 2.41 |
| QX-PO/IDTBR | -4.96 | -2.69 | 2.27 |
| QX-PS/IDTBR | -5.02 | -2.66 | 2.36 |

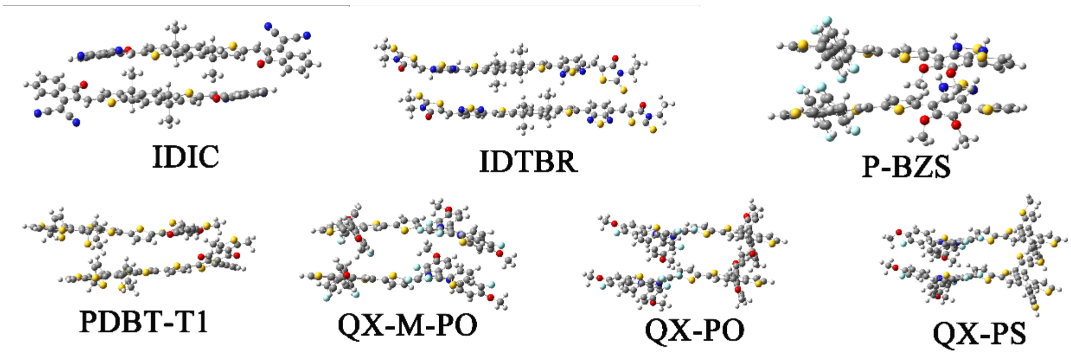


**Fig S1. The optimized face-to-face dimer structures of IDIC, IDTBR and five kind ofpolymers.**

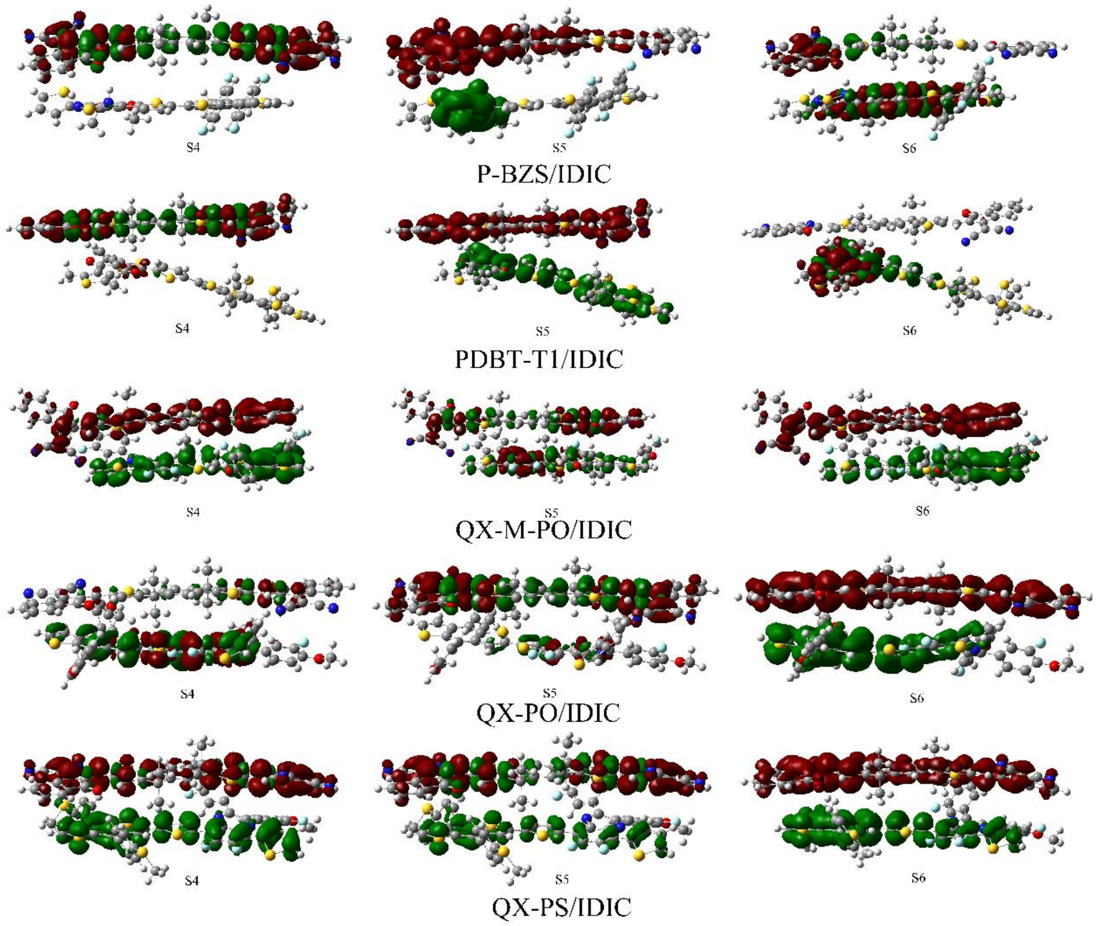


**Fig S2. Charge density difference (CDD) plots of polymer/IDIC (S4-S6); where red represents electrons and green represents holes.**

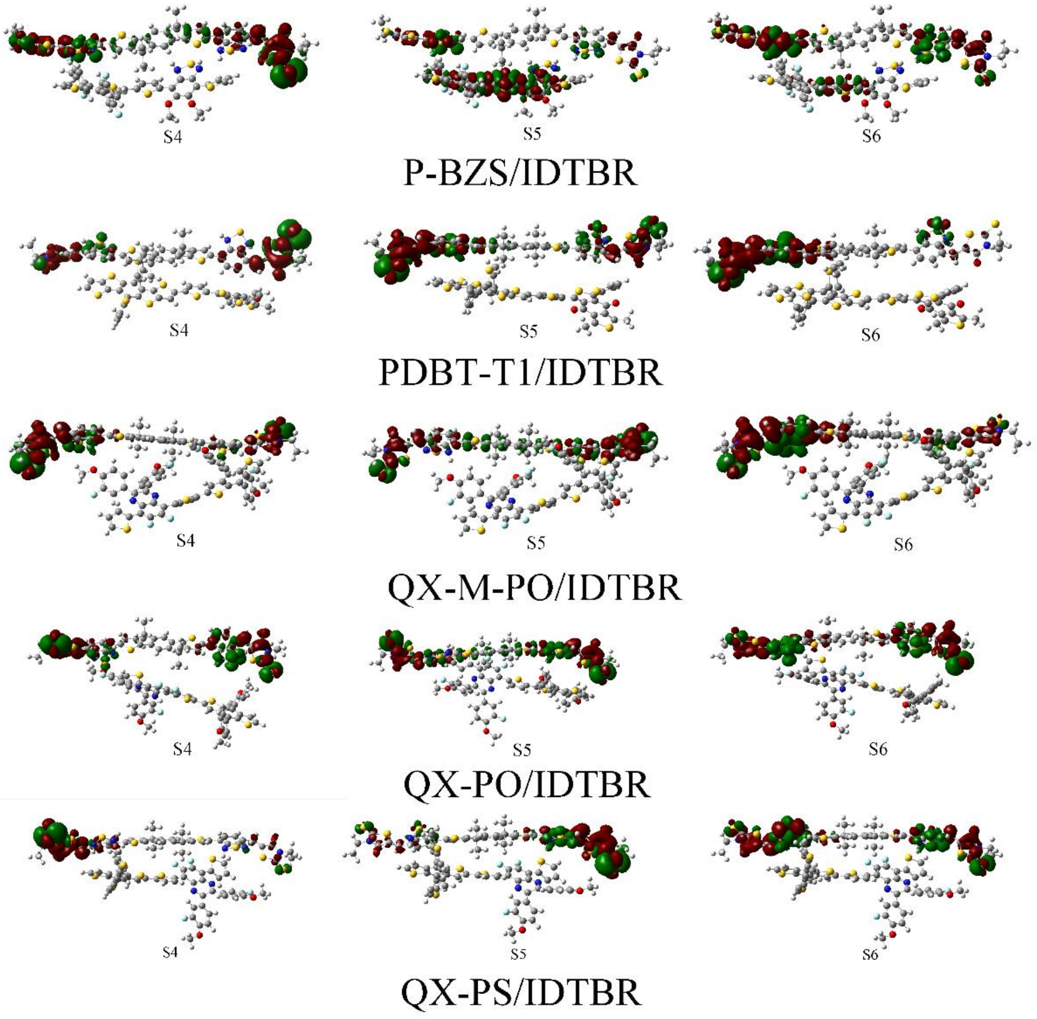


**Fig S3. Charge difference densities (CDD) plots of D/A interfaces polymer/IDTBR (S4-S6); where red represents electrons and green represents holes.**
